# EOAI, a ubiquitin-specific peptidase 5 inhibitor, prevents non-small cell lung cancer progression by inducing DNA damage

**DOI:** 10.1186/s12885-023-10506-0

**Published:** 2023-01-07

**Authors:** Yuanyuan Zheng, Longhao Wang, Xiaoyu Niu, Yongjun Guo, Jiuzhou Zhao, Lifeng Li, Jie Zhao

**Affiliations:** 1 Internet Medical and System Applications of National Engineering Laboratory, the First Affiliated Hospital of Zhengzhou University, Zhengzhou, 450052 Henan China; 2 Department of Anesthesiology, the Affiliated Cancer Hospital of Zhengzhou University, Henan Cancer Hospital, Zhengzhou, 450008 Henan China; 3 Department of Molecular Pathology, the Affiliated Cancer Hospital of Zhengzhou University, Henan Cancer Hospital, Zhengzhou, 450008 Henan China; 4 Henan Key Laboratory of Molecular Pathology, Zhengzhou, 450008 Henan China; 5 Department of Pharmacy, the First Affiliated Hospital of Zhengzhou University, Zhengzhou, 450052 Henan China

**Keywords:** Non-small cell lung cancer, DNA damage, USP5 inhibitor, Apoptosis, Autophagy

## Abstract

**Objective:**

Targeting deubiquitinases (DUBs) has emerged as a promising avenue for anticancer drug development. However, the effect and mechanism of pan-DUB inhibitor EOAI on non-small cell lung cancer (NSCLC) remains to be studied.

**Materials and methods:**

The expression of ubiquitin-specific peptidase 5 (USP5) in NSCLC was evaluated by immunohistochemistry. The effect of the USP5 inhibitor, EOAI, on NSCLC cell growth and cell cycle was evaluated by CCK-8 and PI staining. Apoptosis was detected by Annexin V-FITC/PI double staining. Autophagy was examined by LC3 immunofluorescence. Comet assay and γ-H2AX immunofluorescence staining were used to detect DNA damage, and Western blotting was used to detect the expression of apoptosis, cycle, autophagy and DNA damage-related proteins. In vivo experiments demonstrated the effect of EOAI on NSCLC.

**Results:**

We also found that USP5 was significantly upregulated in NSCLC tissues in this study. In addition, we show that EOAI can cause DNA damage in NSCLC cells while modulating the transcriptional activity of P53, thereby inducing cell cycle arrest in NSCLC cells, autophagy and apoptosis. In vivo experiments have shown that EOAI can inhibit tumors and synergistically enhance the anti-tumor effect of cisplatin.

**Conclusion:**

USP5-mediated epigenetic regulation of oncogenes promotes the occurrence of NSCLC, which provides ideas for developing potential targeted therapy.

**Supplementary Information:**

The online version contains supplementary material available at 10.1186/s12885-023-10506-0.

## Introduction

Lung cancer is the most common cause of cancer-related death worldwide, accounting for 1.8 million deaths in 2020 [1]. The development of treatments for non-small cell lung cancer (NSCLC) has been hindered over the past 60 years by disease heterogeneity, complications, and the lack of safe, effective drug therapies [2]. Although immunotherapy and targeted therapy have significantly improved the efficacy of NSCLC treatment in the past 20 years, most NSCLC patients develop resistance to targeted drugs and disease progression at advanced stages [3]. Therefore, it is crucial to investigate new molecular markers and develop potential therapeutic targets for the treatment of NSCLC in the future.

Almost all biological processes are controlled by the conjugation of ubiquitin with the post-translational modification of the target protein, so the research on protein ubiquitination continues to expand. Since ubiquitin controls protein stability through the action of hundreds of enzymes, using the ubiquitin system to target specific enzymes and thereby reshape the proteome holds great promise for improving disease prognosis [4]. USP5 belongs to a ubiquitin-specific protease family of deubiquitinases (DUBs) and is associated with various diseases, including cancer. USP5 prefers to cleave unanchored (not coupled to a target protein) polyubiquitin chains while also being able to remove polyubiquitin chains from protein substrates, so it is essential for free ubiquitin recycling [5]. In addition, USP5 is also critical for maintaining the steady state of the monoubiquitin pool. Like other USP family deubiquitination enzymes, USP5 is involved in the occurrence and development of various cancers. It has been reported that USP5 overexpressed in many tumors and implicated in its progression, including pancreatic cancer [6], breast cancer [7], and glioblastoma [8]. Wang S et al. found that USP5 protein has five key domains, including the implicit ZnF domain and c-box domain, that interact with c-Maf, demonstrating that c-Maf is a crucial factor in USP5-mediated myeloma cell proliferation and survival [9]. Liu Y et al. found that the Histidine-rich protein Hpn activates the p14-p53 pathway by inhibiting USP5 and promoting the apoptosis of liver cancer cells [10]. In addition, studies have shown that USP5 is significantly up-regulated in pancreatic ductal adenocarcinoma (PDAC), and inhibition of USP5 can significantly reduce the growth of PDAC cells, suggesting that USP5 plays a vital role in the occurrence and development of pancreatic cancer [11]. The above studies show that USP5 plays an essential role in the process of various cancers and is an attractive anticancer target. Therefore, therapies targeting USP5 may play a positive role in cancer treatment.

Several studies have shown the effect of USP5 on NSCLC, including promoting the proliferation of NSCLC cells and stabilizing the expression of PD-L1 [12, 13]. While in our study, we found that USP5 was highly expressed in NSCLC tissues and was associated with poor prognosis in patients. Further investigations discovered that by inhibiting USP5 with EOAI, NSCLC cells underwent DNA damage and subsequently caused activation of the p53 transcription factor, which caused cell cycle arrest, and induced apoptosis and autophagy in NSCLC cells. We investigated the clinical significance of USP5 in NSCLC and revealed the molecular mechanism of targeting USP5 to treat NSCLC. Our findings provide evidence that reshaping the cancer proteomic landscape using innate degradation mechanisms may impact cancer treatment outcomes and provide new insights into molecularly targeted therapies for NSCLC.

## Materials and methods

### Antibody and reagents

The primary antibodies used were as follows: total poly ADP-ribose polymerase (PARP) (No. 5625), Bax (No. 5023), Bak (No. 12105), Survivin (No. 2808), cleaved Caspase 3 (No. 9664), cleaved Caspase 9(No.7237), Mcl-1(No. 94296), Bcl-xl (No. 2764), XIAP (No. 2045), c-IAP1(No. 7065), cyclinB1(No. 4138), p-H3(Ser10) (No. 53348), ATF4(No. 11815), c-Myc(No. 18583), LC3(No. 12741), γ-H2AX(No. 9718), Ki-67 (No. 9449), (Cell Signaling Technology, Inc., Boston, MA), P21(No. AP021), P27(No. AP027), Cleaved PARP (No. AF1567), AMPKα(No. AF6195) p-AMPKα (Thr172) (No. AA393), P53(No. AF1162), p-P53(No. AF5893), caspase-9 ( No. AC062), caspase-3 ()(Beyotime, China), Bcl-2 (No. ab32124), USP5 (No. ab154170), Noxa (No. ab13654), FOX3A (No.ab109629), (Abcam Trading Company Ltd), HIF1α (No. 20960–1-AP), c-MYC (No. 67447–1-Ig), p-mTOR (Ser2448) (No. 67778–1-Ig) (Proteintech, Wuhan, China). GAPDH (Hangzhou Goodhere Biotechnology, China) was used as the loading control. All the primary antibodies were diluted in 1:1000 and incubated at 4˚C overnight. The secondary antibodies are peroxidase-conjugated goat anti-mouse IgG and peroxidase-conjugated goat anti-rabbit IgG (diluted in 1:5000, ZGSB. Bio, Inc., Beijing, China). EOAI (No.HY-111408), 3-Methyladenine, (3MA, No. HY-19312), and Pifithrin-β, (PFT, No. HY-16702), Cisplatin, (CDDP, No. HY-17394) were purchased from Med Chem Express (MCE).

### Immunohistochemistry (IHC)

USP5 expression was assessed in neoplastic cells and peritumoral tissue. The human NSCLC tissue array (HLugA180Su07) was bought from Shanghai Outdo Biotech Co. Ltd. Tumor tissue was dehydrated and incubated with peroxidase. Antigen retrieval was then performed in a pressure cooker using 0.01 mol/L citrate buffer (pH 6.0). Incubate USP5 antibody (dilution: 1:200), γ-H2AX (dilution: 1:200), Ki-67 (dilution: 1:1000) overnight at 4 °C according to the instructions. The slides were then stained with a Histostain-Plus kit (SP-9000) and 3,3-diaminobenzidine tetrahydrochloride (DAB) (ZLI-9032) (ZSGB-BIO, Beijing, China). Finally, nuclei were stained with hematoxylin. The IHC results were scored by two senior pathologists in a double-blind manner. The following proportion scores were assigned to the sections: 0 if 0% -5% of tumor cells exhibited positive staining, 1 for 6%-25% positive cells, 2 for 26%–50% positive cells, 3 for 51%–75% positive cells, 4 for 76%–100% positive cells. In addition, the staining was scored on a scale of 0–3: 0, negative; 1, weak; 2, moderate; and 3, strong. The positive grade of IHC was determined according to the positive proportion of tumor cells and staining intensity: 0 was classified as negative (-), 1–3 as weakly positive ( +), 4–5 as positive (+ +), and 6–7 as strongly positive (+ + +). Survival analysis was performed according to USP5 expression, with low USP5 expression including (-) and ( +), and high expression including (+ +) and (+ +  + +).

### Cell lines and culture conditions

NSCLC cell lines (A549, H460, Cslu-3, H3122) and Normal lung epithelial cell line (BEAS-2B) were purchased from the Shanghai Cell Resource Center Academy of Sciences. All cell lines were tested by STR (QuiCell Biological, Shanghai, China) and showed no cross-contamination of cell lines. Cells were cultured in a complete medium containing 90% DMEM (No. 10–013-CVRV, CORNING, China) with 4 mM glutamine (No. 2323081, Glibco) and 1% penicillin–streptomycin (No.C0224, Beyotime, China) and 10% fetal bovine serum (No. FSP500, ExCell Bio) in a 37 °C cell incubator with 5% CO_2_. Cells were passaged every 2–3 days, and cells in the logarithmic growth phase were selected for experiments.

### Immunoblotting

Cells were incubated with DMSO or EOAI. After 48 h, cells were lysed in the RIPA lysis buffer containing 150 mM NaCl, 50 mM Tris–HCl pH 7.5, 1% NP-40, 1 mM permethylsulfonate, 1 mM orthovanadate, 1% SDS, 1% protease inhibitors at 4 C. Cell extracts were subject to centrifugation to remove insoluble materials. Total protein concentration was determined by BCA assay (No. P0011, Beyotime, China), and the protein was boiled at 95℃ for 5 min. Proteins were submitted to SDS-PAGE and then transferred onto a polyvinylidene fluoride (PVDF) membranes (No. IPVH00010, Millipore, Germany) by electroblotting. The saturation was done in TBS-Tween 0.1% containing 5% bovine serum albumin, 3% skimmed milk (for anti-TrkA), or 0.2% casein (for anti-sortilin) for 1 h at room temperature. Membranes were incubated with primary antibodies in saturation buffer (overnight, 4 °C). After being washed with TrisBuffered Saline Tween-20 (TBST), the membrane was incubated with secondary antibodies for 2 h at room temperature. ECL Kit (No. P0018, Beyotime, China) and Automatic chemiluminescence image analyzer (Tanon5200, Shanghai, China) visualized the proteins.

### Cell proliferation and cell colony-forming unit assay

For cell proliferation assay, A549, H460, Calu-3, and H3122 cells in the logarithmic growth phase were seeded in 96-well with 3000–5000 cells per well. Cells were treated with EOAI dissolved in DMSO and prepared at different concentrations (1, 1.5, 2, 2.5, 3, 3.5, 4, 4.5 μmol/L) for 48 h. DMSO-treated cells (0.1%) were used as the control group. Then, CCK-8 reagent (No.IV08, Invigentech, USA) was added to each well and incubated at 37 °C for 1 h, and a microplate reader measured the absorbance at 450 nm to quantify cell viability. For the colony-forming unit assay, cells were seeded in 6-well plates at 500–1000 cells per well. DMSO or EOAI was added 24 h after the cells adhered and cultured for another 7–10 days. After washing with phosphate-buffered saline (PBS; No. D8537, Sigma), the cells were fixed with 4% paraformaldehyde (No. P1110, Solarbio, China) for 15 min, then stained with crystal violet solution (No. G1063, Solarbio, China) for 15 min, and counted after washing.

### Cell cycle analysis

Cells after EOAI treatment for 24 h were collected with 0.05% trypsin without EDTA solution and fixed overnight at 4 °C by adding 70% ethanol. After centrifuging, cell suspension with PBS containing 50 μg/ml RNase A and 10 μg/ml PI was kept at 37 °C in the dark. The cell cycle was evaluated by fluorescence activating cell sorter (Becton Dickinson FACScan; Becton–Dickinson, San Jose, CA, USA). The results of flow cytometry were analyzed by ModFit LT 3.1 software.

### Cell apoptosis assay

The NSCLC cells treated with DMSO and EOAI for 48 h were collected and stained with Annexin V-FITC/PI at RT for 15 min in the dark, and then the apoptosis ratio was detected by flow cytometry. The cells were collected similarly, and the Casp-GLOW Fluorescein Active Caspase 3 Staining kit (BioVision, Inc., Milpitas, CA, USA) was selected to detect the activity of Caspase 3.

### Observation of mitochondrial membrane potential

The cells (1 × 10^6^/ml) were inoculated into 6-well plates and treated with different concentrations of EOAI and 0.1% DMSO for 24 h. Mitochondrial membrane depolarization was detected with the mitochondrial membrane potential assay kit with JC-1 according to the manufacturer's protocol (Yeasen, Shanghai, China). The data were acquired and analyzed by flow cytometry. Cells with intact mitochondria displayed high red fluorescence and appeared in the upper right quadrant of the scatterplots.

### Gene knockdown using siRNA

Cells plated in 6-well plates were transfected with two different sequences of siRNAs targeting Noxa and Control using jetPRIME transfection reagent (No. 23Y2307M9, Polyplus-transfection, SA) when 50% confluent. Cells were then incubated with a transfection mixture of 100 nM siRNA and jetPRIME for 24 h. Cells transfected with Noxa and control were used for Cell apoptosis assay or Immunoblotting. All siRNAs were synthesized in Shanghai GenePharma Co. Ltd, China: siNoxa: 5’-GUAAUUAUUGACACAUUUC-3’; siControl:5’- UUCUCCGAACGUGUCACGU-3’.

### Immunofluorescence

The cells were seeded in a petri dish, and after the cells adhered, they were treated with DMSO or EOAI for 24 h. Discarded the medium, washed twice with PBS, and added 4% paraformaldehyde to fix the cells for 20 min, then added anhydrous methanol and placed at -20 °C for 10 min to infiltrate. After blocking with 5% BSA for 20 min at room temperature, LC3/γ-H2AX (1:200) primary antibody was added separately at 4 °C overnight. After that, Alexa Fluor® 488 Goat anti-Rabbit secondary antibody (green, dilution:1:500) (Beyotime, China) was added for 2 h at RT. Finally, the nuclei were labeled with DAPI (5 μg/ml, Beyotime, China, blue) and placed at RT for 20 min. Photographs were taken with a fluorescence microscope (Olympus CKX53).

### Comet assay

Cells were incubated with 0.1% DMSO or EOAI for one day. Mix 20uL of the cell suspension with 100uL of 0.5% low melting point agarose solution and spread it on a solidified 0.5% standard melting point agarose-coated glass slide. Then cover with a cover slip and cure at 4 °C for 30 min. The slides were placed in lysis buffer (2.5 mol/L NaCl, 100 mmol/L EDTA, 10 mmol/L Trizma base, 10% DMSO, and 1% Triton X-100) at 4 °C for 2 h. Under dark conditions, place the slide in the electrophoresis tank, 300 mA, 20 min. The microscope slides were rinsed with 0.4 M Tris HCl and stained with PI (25 μg/ml) for 10 min, and observed the comets and photographed under a fluorescence microscope (Olympus CKX53).

### Xenograft model in C57 mice

Xenograft model in C57 mice 6–8 weeks-old specific pathogen free (SPF) male mice (Beijing Vital River Laboratory Animal Technology Co. Ltd, China) weighing 17-19 g, were randomly divided into four groups with 5 mice in each group. Under the pathogen-free environment, all animals were cared for and anesthetized before experimentation following the Guide for the Care and Use of Laboratory Animals. A mouse subcutaneous lung cancer model was established with Lewis lung cell (LLC) suspension (5 × 10^5^ cells in 100ul PBS), and drug (EOAI:15 mg/kg and CDDP:2.5 mg/kg) treatment was given 3 days after the tumor was successfully implanted. Mice were weighed every 3 days, and tumor size was measured using a vernier caliper. The volume of the tumor is calculated based on its long diameter and short diameter. Tumor volume was obtained by the formula: (length × width2)/2. Mice were sacrificed by cervical dislocation on the 21^st^ day after cell implantation. The tumors were collected for IHC analysis.

### Quantification and statistical analysis

All data were performed in at least three independent experiments. To quantify the cell colony-forming unit assay, count the number of cells in the dish using ImageJ software. Data were shown as mean SD, and error bars represented the standard deviations from three independent experiments. Data graphics and descriptive statistics were presented by using Graphpad prism 8, SPSS 25.0 and the Microsoft Excel data analysis package. The significance between the two groups was obtained using the Student’s *t*-test. Overall survival (OS) was defined as the time from the data of diagnosis to death or the last follow-up examination. Survival curves were performed by the Kaplan–Meier method, and groups were compared using log-rank tests. ns, not significant; *, *p* < 0.05; **, *p* < 0.01; ***, *p* < 0.001.

## Results

### USP5 overexpressed in NSCLC and high USP5 expression predicted poor prognosis

**Fig. 1:**
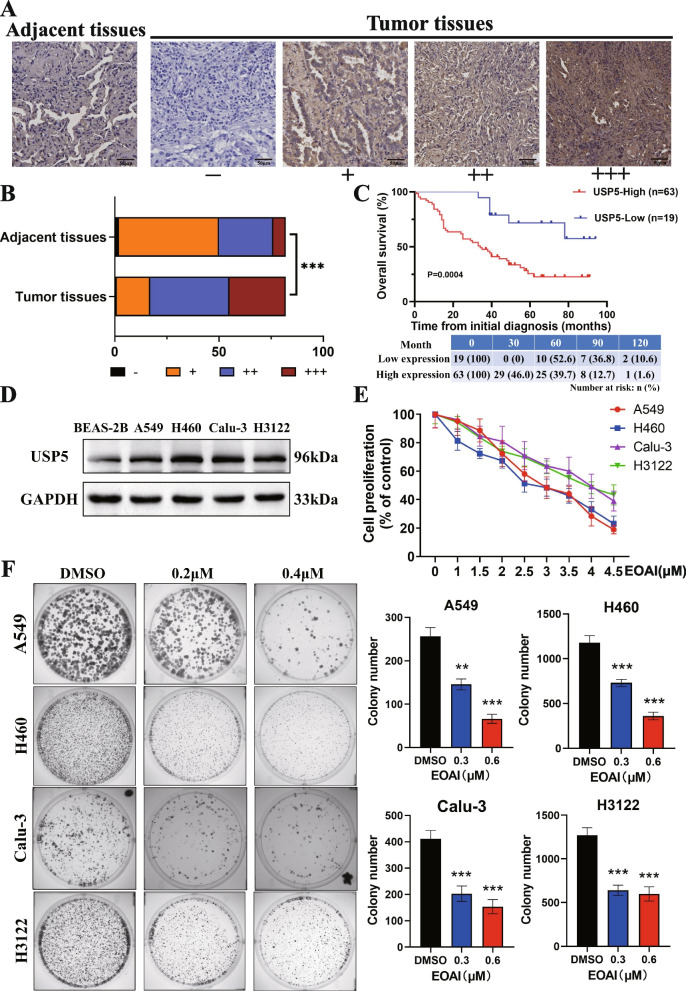
USP5 is highly expressed in lung cancer, and its inhibitor, EOAI, can inhibit NSCLC cell growth and colony formation. **A** USP5 overexpressed in NSCLC tissues. Representative pictures of IHC staining for USP5 from the weakest (− , group 1) to the strongest (+ +  + , group 4) based on increasing staining intensity. **B** Classification of tumor samples according to IHC staining (*n* = 82). The statistical significance of differences between groups was analyzed by the chi-square test of SPSS software (*P* < 0.01). **C** Effect of USP5 expression level on survival of NSCLC patients. Patients with high USP5 expression had a worse prognosis than those with low USP5 expression (*P* = 0.0004). **D** USP5 is highly expressed in NSCLC cells. By contrast, USP5 expression was up-regulated in NSCLC cells compared with normal non-epithelial cells. **E** Examination of the effect of EOAI on NSCLC cells cell viability. Cells were treated with DMSO or EOAI for 48 h, and viability was detected by CCK-8 kit. **F** Detection of the efficacy of EOAI on colony formation in NSCLC cells. NSCLC cells were treated with EOAI as indicated for 10 days, then fixed, stained, and counted. Cell colonies are shown on the left panel, and then the number of cell colonies was statistically analyzed as shown on the right panel. The t-test of GraphPad Prism8 software assessed the statistical significance of differences between groups in panels D and F. * Represents the difference between the two groups was significant (*P* < 0.05). All data represent at least three independent experiments (*n* = 3; error bar, SD)

To determine the expression of USP5 in NSCLC tissues, we performed immunohistochemical staining in tissue microarray with NSCLC and adjacent tissues. The results showed that the expression of USP5 was increased in NSCLC tissues compared to peritumor samples (Fig. 1A). Eighty-two lung cancer patients were divided into 4 groups according to the positive staining ratio of cancer cells. After the chi-square test, the expression of USP5 in tumor tissue was higher than that in adjacent tissue, and the difference was statistically significant (Fig. 1B). The association between USP5 expression and clinicopathological features of 82 NSCLC patients in this study is shown in Table 1. The correlation between USP5 expression and pathological features of patients was determined by the Chi-square testor Fisher’s exact tests, if appropriate. 63 patients (76.8%) had high USP5 expression, and 19 patients (23.2%) had low USP5 expression. The expression of USP5 was not related to gender, age, lymph node metastasis, pathological grade, the EGFR mutation and ALK translocation but was related to tumor size, and the difference was statistically significant. Next, we analyzed the relationship between USP5 and the prognosis of patients with NSCLC. Kaplan–Meier survival analysis revealed that high expression of USP5 was positively associated with poorer prognosis in patients with NSCLC (*P* < 0.05; Fig. 1C). The median OS time for patients with USP5 low and high expression was 58 months and 33 months, respectively. These findings revealed that USP5 has higher expression in lung cancer tissues and may be related to the progression of NSCLC.

**Table 1:** Association between the expression of USP5 and clinicopathological parameters in NSCLC

Parameter	USP5 Expression		*P*-value
	Low (0–1)	High (2–3)	
All case	19 (23.2%)	63 (76.8%)	
Gender			0.5570
Male	12 (63.2%)	35 (55.6%)	
Female	7 (36.8%)	28 (44.4%)	
Age			0.6727
< 60	11 (57.9%)	33 (52.4%)	
≥ 60	8 (42.1%)	30 (47.6%)	
Pathological grading			0.6103
I	2 (10.5%)	6 (9.5%)	
II	12 (63.2%)	31 (49.2%)	
III	5 (26.3%)	26 (41.3%)	
Tumor size (T)			0.0252^a^
1	8 (42.1%)	8 (12.7%)	
2	9 (47.4%)	35 (55.6%)	
3	2 (10.5%)	16 (25.4%)	
4	0 (0%)	4 (6.3%)	
EGFR			0.5278
Negative	12 (92.3%)	40 (80.0%)	
Positive	1 (7.9%)	10 (20.0%)	
ALK			0.8694
Negative	11 (78.6%)	43 (81.1%)	
Positive	3 (21.4%)	10 (18.9%)	

*EGFR* Epidermal growth factor receptor. *ALK* Anaplastic lymphoma kinase

^a^ Statistically significant *p*-values ( *p* < 0.05 using chi-square test or Fisher exact tests, if appropriate)

### EOAI inhibits the proliferation of NSCLC cells

Cell proliferation assay and colony formation assay were used to verify the effect of EOAI on the proliferation of NSCLC cells, which showed the inhibition of USP5 caused profoundly reduced proliferation of NSCLC cells (Fig. 1D). Colony experiments showed the same trend that EOAI could inhibit the proliferation of NSCLC cells (Fig. 1E, F). EOAI has similar growth inhibitory effects on A549, H460, Calu-3, and H3122, so we selected A549 and H460 for the study.

### EOAI induces G2/M arrest of NSCLC cells

**Fig. 2:**
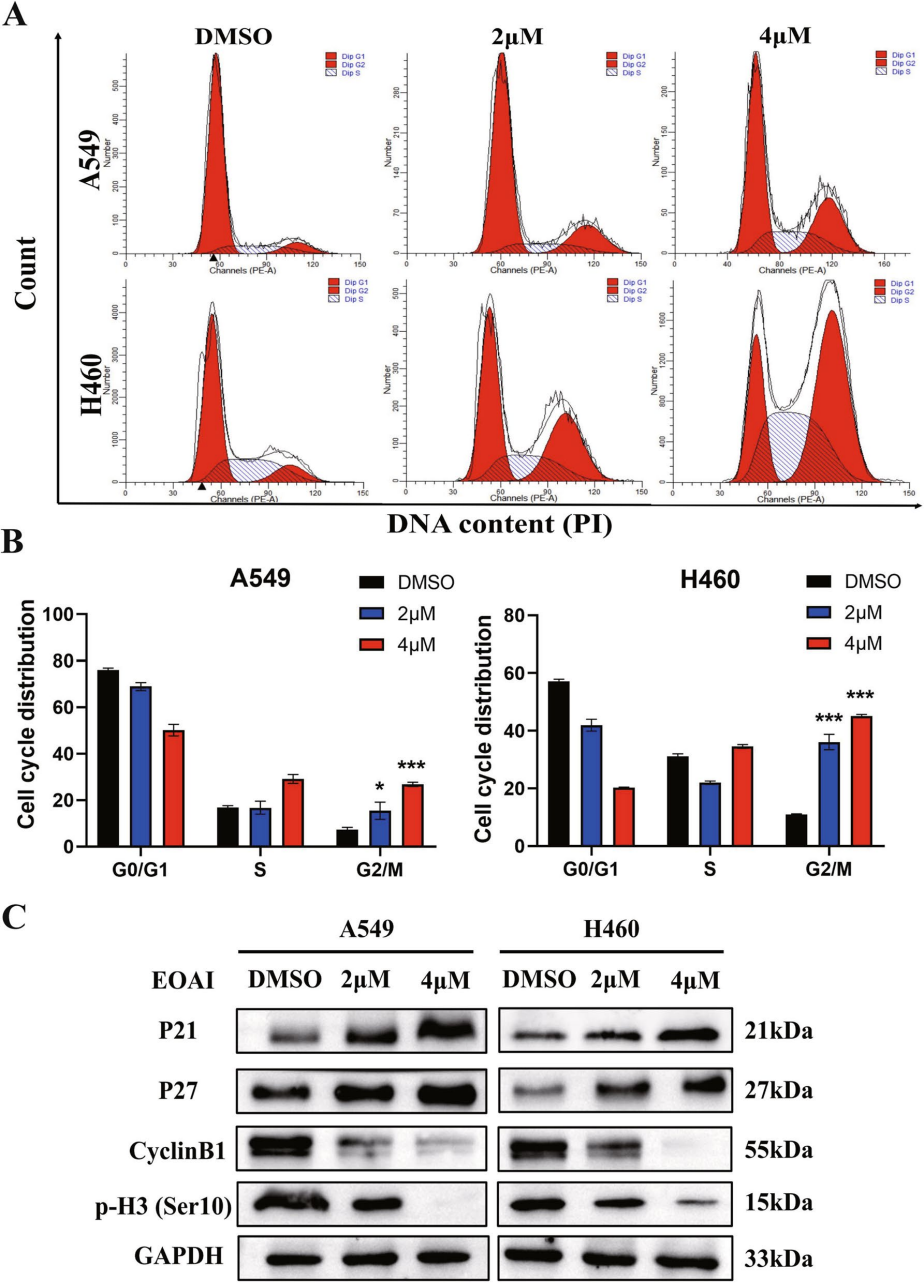
EOAI triggered G2/M cell cycle arrest in NSCLC cells. A549 and H460 cells were treated with EOAI as indicated for 24 h, followed by PI staining. Representative pictures were shown in panel (**A**). Distribution was analyzed and shown in panel (**B**). The t-test of GraphPad Prism8 software assessed the statistical significance of differences between groups in panel b. * The difference between the two groups was significant (*P* < 0.05). All data represent at least three independent experiments (*n* = 3; error bar, SD). **C** The expression of cell-cycle-related proteins was detected. A549 and H460 cells were subsequently treated with EOAI at indicated concentrations and subjected to western blotting. Use GAPDH as the loading control. All data were representative of at least three independent experiments

To further clarify the mechanisms underlying the inhibition of cell proliferation by EOAI, we detected the cell cycle changes of NSCLC cells after EOAI treatment by flow cytometry. As shown in Fig. 2A and B, cells were at the G2/M phase after being treated with EOAI, which were profoundly higher than those of control cells. The changes in cell cycle-related protein levels were detected by Western blotting after EOAI treatment. It was found that P21 and P27 were up-regulated (Fig. 2C). Meanwhile, EOAI can down-regulate the landmark protein p-H3 in mitosis phase [14] and cyclinB1 in G2/M phase [15] in a concentration-dependent manner (Fig. 2C). The above results indicate that EOAI can induce G2/M phase arrest in NSCLC cells.

### Noxa plays an important role in EOAI-triggered intrinsic apoptosis

**Fig. 3:**
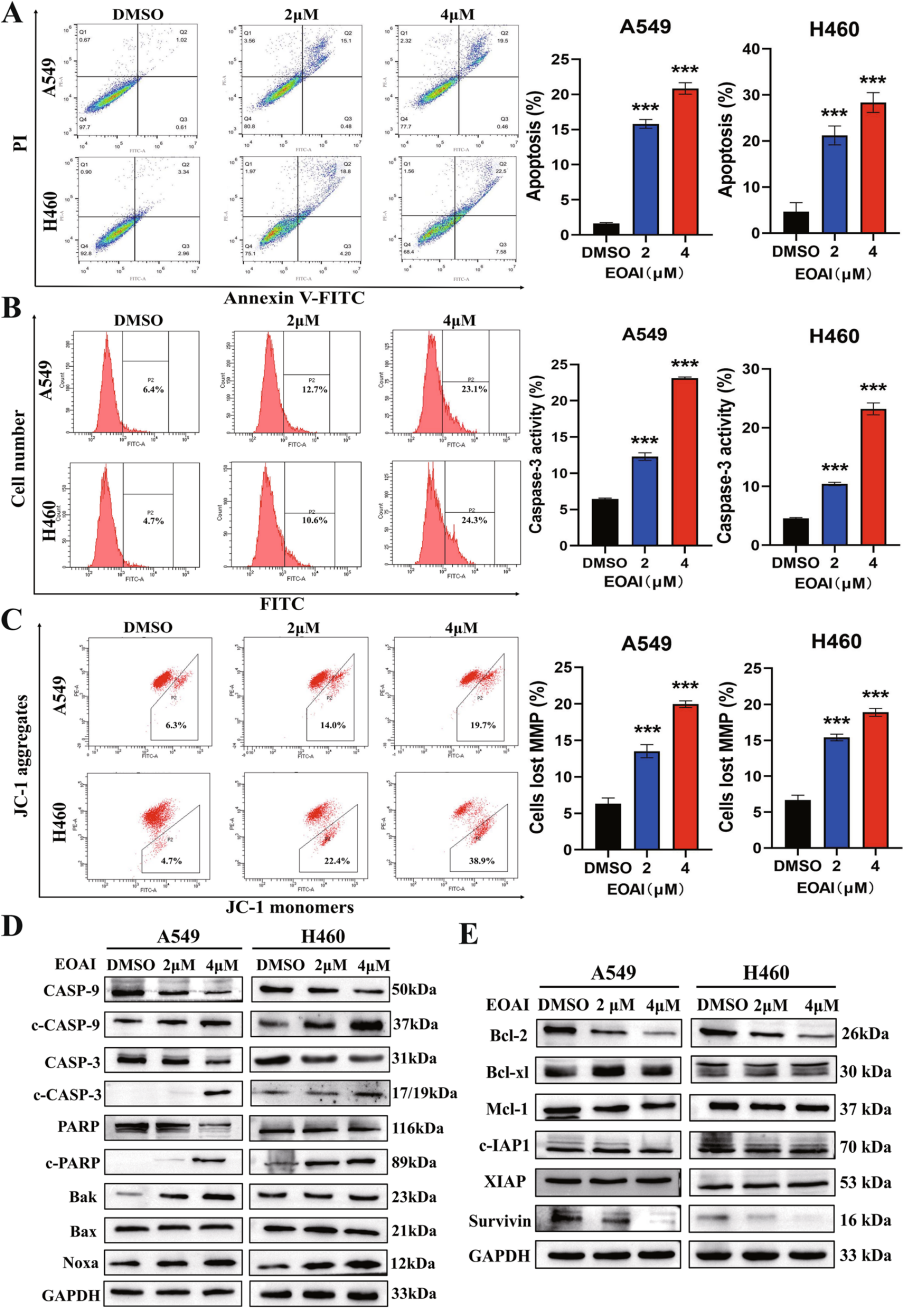
EOAI induces intrinsic apoptosis. **A** A549 and H460 cells were treated with EOAI for 48 h. Annexin V-FITC/PI double-staining determined apoptosis. Representative FACS pictures are shown in the left panel. The cells in the second (upper right) and fourth (lower right) quadrants of the scatter plots were Annexin V + . The percentage of Annexin V + cells was defined as apoptosis rate and shown in the right panel. **B** A549 and H460 cells were treated with EOAI for 48 h. CASP3 activity was examined by FACS (left panel). The percentage of cells with activated caspase3 is shown in the right panel. **C** A549 and H460 cells were treated with EOAI for 24 h. mitochondrial membrane potential (MMP) was monitored with JC-1 dye (left panel). Cells that lost MMP appeared in the right lower quadrant and were analyzed in the right panel. The statistical significance of differences between groups in panels (**A**, **B**, and **C**) was assessed by the t-test of GraphPad Prism8 software. * The difference between the two groups was significant (*P* < 0.05). All data represent at least three independent experiments (*n* = 3; error bar, SD). **D** Detection of the expression of apoptotic, proapoptotic, and anti-apoptotic proteins after EOAI treatment for 48 h. A549 and H460 cells were treated with EOAI, and cell extracts were prepared for western blotting analysis. GAPDH and β-actin were used as the loading control. All data were representative of at least three independent experiments

We next investigated whether cell apoptosis was responsible for the growth-inhibitory effects of EOAI. This hypothesis was validated after we observed that EOAI significantly increased Annexin V^+^ cells (Fig. 3A) and caspase-3 (CASP3)-activated cells (Fig. 3B). It is well known that early apoptosis of cells is related to the production of ΔΨm during the action of the mitochondrial respiratory chain. ΔΨm is often employed as an indicator of cell viability to determine whether cells have undergone apoptosis [16]. The results showed that EOAI caused the loss of mitochondrial membrane potential (MMP) in NSCLC cells (Fig. 3C). Subsequently, we detected apoptotic proteins (Bak, Bax, and Noxa) (Fig. 3D) and anti-apoptotic proteins (Bcl-2, Bcl-cl, Mcl-1, c-IAP1, XIAP, Survivin) (Fig. 3E) in EOAI-treated cells. It was found that among these proteins, the pro-apoptotic protein Noxa was significantly induced.

### EOAI activates Noxa to induce intrinsic apoptosis

**Fig. 4:**
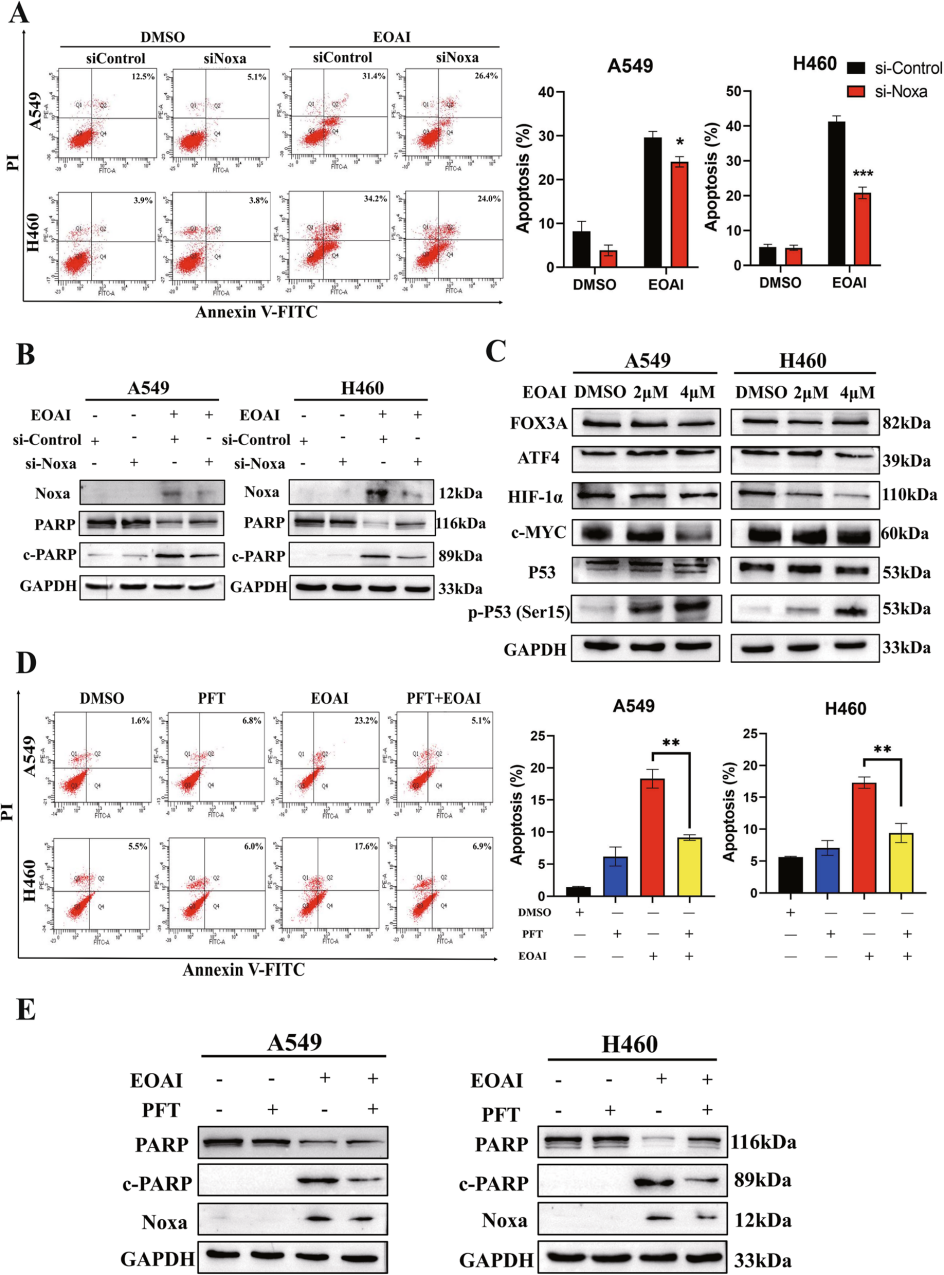
p-P53-Noxa was responsible for EOAI-induced apoptosis in NSCLC cells. Noxa was critical for apoptosis induced by EOAI in NSCLC cells. After being transfected with the control siRNA or Noxa siRNA, A549 and H460 cells were treated with EOAI (2 μΜ) for 48 h. **A** Apoptosis was quantified by Annexin V-FITC/PI double-staining analysis (left panel), and Annexin V + cell populations were defined as apoptosis (right panel). **B** The efficiency of siNoxa and its effect on the level of cleaved PARP. A549 and H460 cells were treated as described in panel A, and cell proteins were extracted for Western blotting analysis. GAPDH was used as the loading control. **C** The screen of Noxa-related transcription factors. A549 and H460 cells were treated with DMSO or EOAI, and cell lysates were measured by Western blotting with specific antibodies. GAPDH was used as the loading control. **D** The expression of P53 was responsible for EOAI- induced apoptosis in NSCLC cells. After transfected with the inhibitor of P53, Pifithrin-β (PFT), A549, and H460 cells were further treated with EOAI (2 μΜ) for 48 h. Apoptosis was examined by Annexin V-FITC/PI double-staining analysis (left panel), and Annexin V + cell populations were defined as apoptosis (right panel). **E** The knockdown efficiency of PFT and its effect on the expression of Noxa and cleaved PARP were measured by Western blotting analysis. GAPDH was used as the loading control

Noxa was first reported as the p53-induced pro-apoptotic gene in 2000, and blocking endogenous Noxa induction resulted in the inhibition of apoptosis [17]. In our study, it was observed that EOAI-induced apoptosis could be rescued after silencing of Noxa (Fig. 4A). In addition, downregulation of Noxa also resulted in reduced cleaved PARP by siRNA silencing (Fig. 4B). This finding highlights the critical role of Noxa in EOAI-induced intrinsic apoptosis. Next, we examined Noxa-regulated transcription factors (TFs) expression levels after EOAI treatment. The results showed that EOAI treatment significantly increased the expression of p53 phosphorylation level (Ser 15) and slightly down-regulated the expression of HIF-1α, while other TFs changed slightly (Fig. 4C). Therefore, we have reason to speculate that EOAI-activated p53 in NSCLC cell lines to induce apoptosis. PFT has been shown to inhibit the transcriptional activity of p53 effectively and simultaneously inhibit the expression of many pro-apoptotic genes [18]. Our results suggested that PFT can reduce EOAI-induced apoptosis, including a reduction in apoptotic cells (Fig. 4D) and apoptotic proteins (Fig. 4E).

### EOAI induces autophagy by activating AMPK

**Fig. 5:**
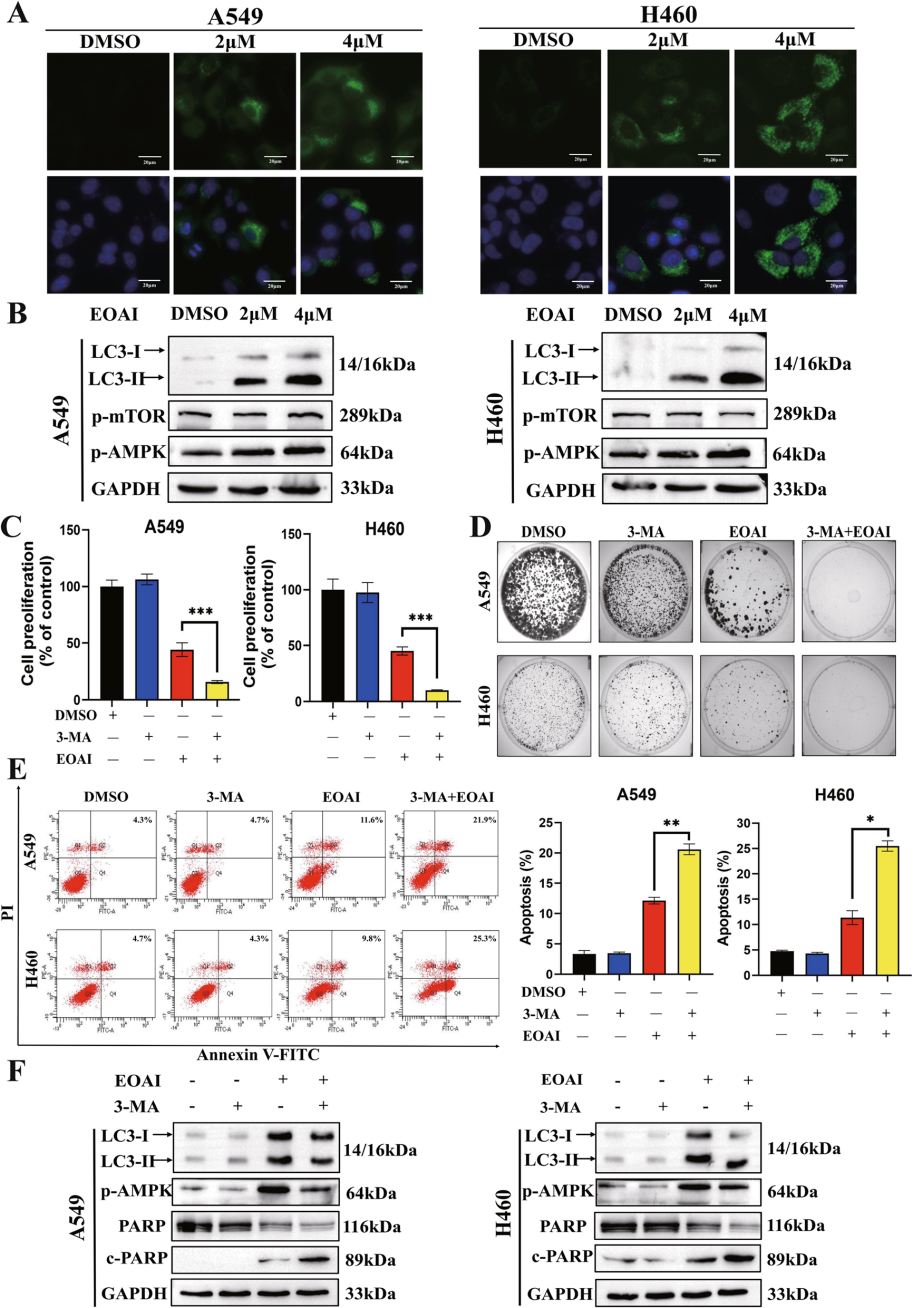
EOAI treatment activated autophagy. **A** Immunofluorescence of LC3. A549 and H460 cells were treated with EOAI (2 μΜ) for 24 h. Cells were then incubated with LC3 primary antibody (1:200, overnight at 4℃) and Alexa Fluor 488 Goat Anti-Rabbit IgG (H + L) secondary antibody (green) (1:500, 2 h at room temperature), respectively. The nuclei were stained by DAPI (blue) (5 μg/mL, 20 min at room temperature). Images were captured using fluorescence microscopy (magnification: 200). Representative images were shown. **B** Detection of the expression of LC3. A549 and H460 cells were treated with EOAI, and cell extracts were prepared for Western blotting analysis. GAPDH was used as the loading control. **C** Blockage of autophagy enhances EOAI-induced cell growth suppression of NSCLC cells. A549 and H460 cells were treated with 2 μmol/L EOAI alone or in combination with 3MA (10 mmol/L). Cell viability was measured using the CCK-8 assay. **D** Detection of the efficacy of EOAI alone or in combination with 3MA on colony formation on NSCLC cells. NSCLC cells were treated with EOAI alone or in combination with 3MA as indicated for 10 days and then fixed and stained. **E** Blockage of the autophagic response increased the EOAI-induced apoptosis of NSCLC cells. A549 and H460 cells were treated with 2 μmol/L EOAI alone or in combination with 3MA (10 mmol/L). Apoptosis was determined by the Annexin V-FITC/PI double-staining analysis. **F** Total PARP and cleaved PARP, and p-AMPK were detected by immunoblotting. GAPDH was used as the loading control. All data were representative of at least three independent experiments

Much evidence suggests that dysregulation of the ubiquitin/proteasome system is strongly associated with autophagy [19, 20]. In our study, LC3 immunofluorescence staining was positive (Fig. 5A), and LC3-I/II protein expression was increased (Fig. 5B) after EOAI treatment, demonstrating the occurrence of autophagy. Furthermore, the up-regulated of phosphorylated AMPK (Fig. 5B) suggested that p-AMPK was involved in EOAI-induced autophagy. Meanwhile, we found that the autophagy inhibitor 3-methyladenine (3-MA) combined with EOAI significantly inhibited NSCLC cell proliferation (Fig. 5C, D), promoted apoptosis (Fig. 5E), inhibited autophagy, and increased the expression of cleaved PARP protein compared with EOAI alone (Fig. 5F).

### EOAI induces DNA damage

**Fig. 6:**
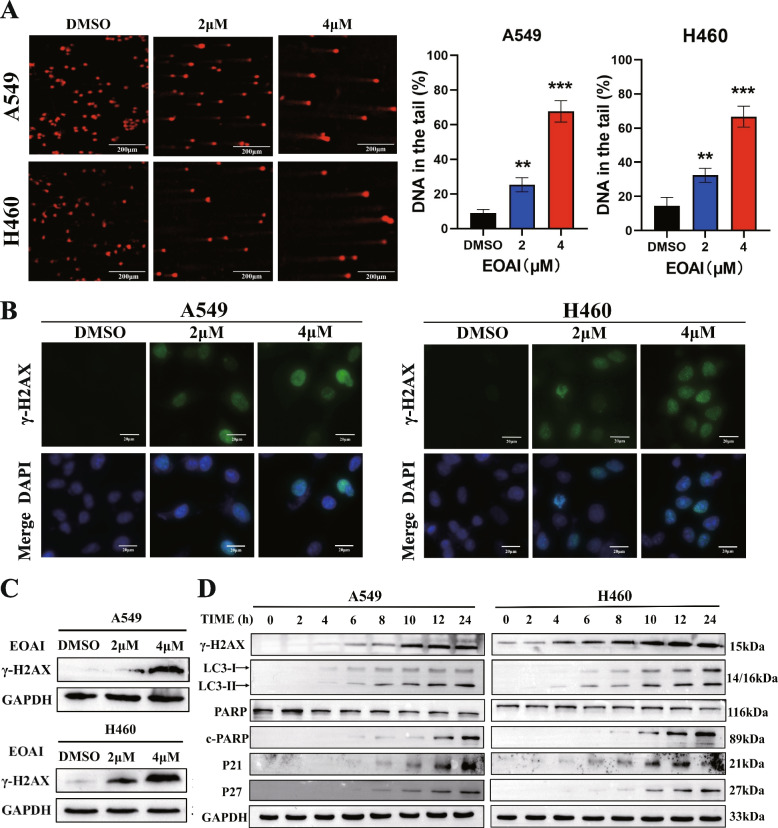
Treatment with EOAI induced DNA damage in NSCLC cells. **A** The comet assay detected DNA damage. A549 and H460 cells were treated with DMSO or EOAI as indicated. 24 h later, cells were collected and detected by the comet assay as described in the Materials and methods section. Images were captured under fluorescence microscopy, represented pictures in the left panel, and the quantization of comet tails is shown in the right panel. **B** γ-H2AX foci were determined by immunofluorescence. A549 and H460 cells were treated with EOAI at indicated concentrations. γ-H2AX foci were determined by immunofluorescence. **C** The expression of γH2AX was determined by western blotting. A549 and H460 cells were treated with EOAI, and cell extracts were subjected to western blotting. GAPDH served as a loading control. **D** Relationship between DNA damage and cell cycle, autophagy, and apoptosis. A549 and H460 cells were treated with EOAI. Cells were collected at different time points and subjected to western blotting. The expression of γ-H2AX, LC3, total PARP and cleaved PARP, p21, p27, and in different time points was detected. GAPDH served as a loading control

DNA damage triggers a series of signaling cascades that promote cell survival, including DNA repair, apoptosis, autophagy, and cycle arrest [21]. γ-H2AX was the first histone modification caused by DNA damage discovered in 1998 and has since been widely recognized as a marker of DNA damage [22]. The results of the comet assay showed that high concentrations of EOAI caused cells to develop longer tails (Fig. 6A). At the same time, EOAI treatment resulted in an increase in γ-H2AX-positive cells (Fig. 6B) and an up-regulation of γ-H2AX protein levels (Fig. 6C). In order to further study the relationship between DNA damage and cell cycle arrest, apoptosis, autophagy, we collected EOAI-treated cells at different time points. Through protein level verification, we found that DNA damage occurred at the earliest time (Fig. 6D).

### Phosphorylated P53 is a key molecule for EOAI to function

**Fig. 7:**
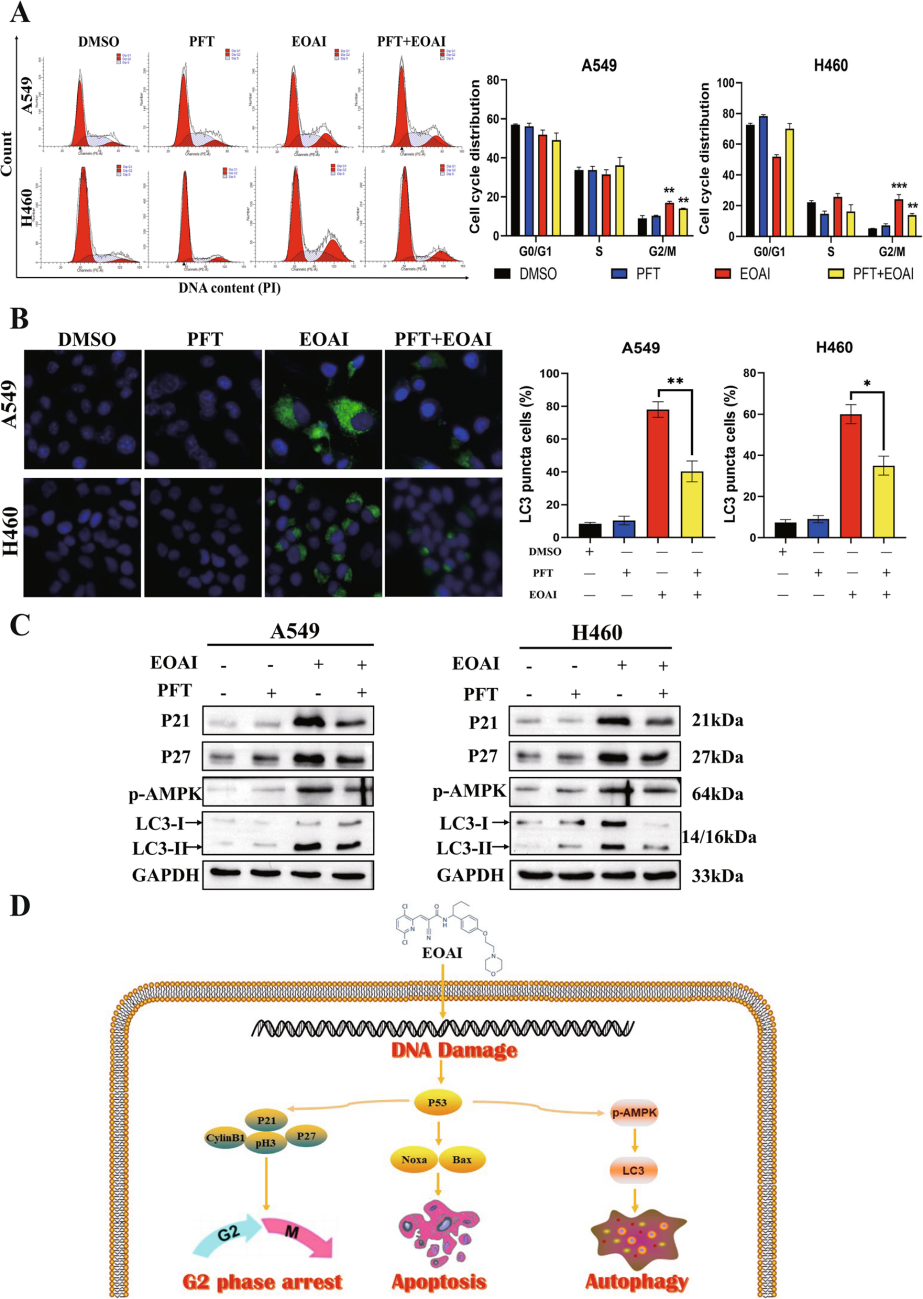
Activation of p53 enables EOAI-induced cell cycle arrest, apoptosis, and autophagy. **A** Inactivation of p53 reduced the extent of G2/M cell cycle arrest. A549 and H460 cells were treated with EOAI single (2 μM) or combined with PFT (10 mM). The cell cycle profile was analyzed by PI staining and FACS, and Modfit and GraphPad Prism8 software analyzed cell distribution. **B** PFT reduced EOAI-induced autophagy. A549 and H460 cells were treated with EOAI single (2 μM) or combined with PFT (10 mM). LC3 was detected using immunofluorescence assay, representative pictures were captured (left panel), and LC3 puncta cells were statistically analyzed (right panel). Statistical significances of differences between groups in right panels of A and B were assessed by the t-test of GraphPad Prism8 software. * Represents a significant difference between the two groups (*P* < 0.05). All data were representative of at least three independent experiments (*n* = 3; error bar, SD). **C** Effect of PFT on protein expression of p21, p27, p-AMPKα, and LC3. A549 and H460 cells were treated with EOAI single (2 μM) or combined with PFT (10 mM). Cell proteins were detected using specific antibodies. GAPDH was used as the loading control. All data were representative of at least three independent experiments. **D** Schema of the mechanism for EOAI-induced cell cycle arrest, apoptosis, and autophagy in NSCLC. EOAI treatment-induced DNA damage and led to G2/M cell cycle arrest by p53-p21/p27 axis; triggered apoptosis through regulating pro-apoptotic proteins including Bax and Noxa; and stimulated autophagy through p53-dependent AMPK activation

We used the P53 transcriptional activity inhibitor PFT to assess whether phosphorylated P53 is equally involved in cycle arrest and autophagy in NSCLC cells. The result showed that PFT could also reverse the cycle arrest caused by EOAI, which has been verified at the cellular levels (Fig. 7A). Likewise, we observed that the effect of EOAI in promoting autophagy (Fig. 7B) was attenuated after the addition of PFT. Such results were also confirmed at the protein level (Fig. 7C). Our results showed that EOAI activated the transcription factor P53 after DNA damage in NSCLC cells, thereby inducing apoptosis, cell cycle arrest, and autophagy (Fig. 7D).

### EOAI synergistically enhanced the antitumor effect of cisplatin

**Fig. 8:**
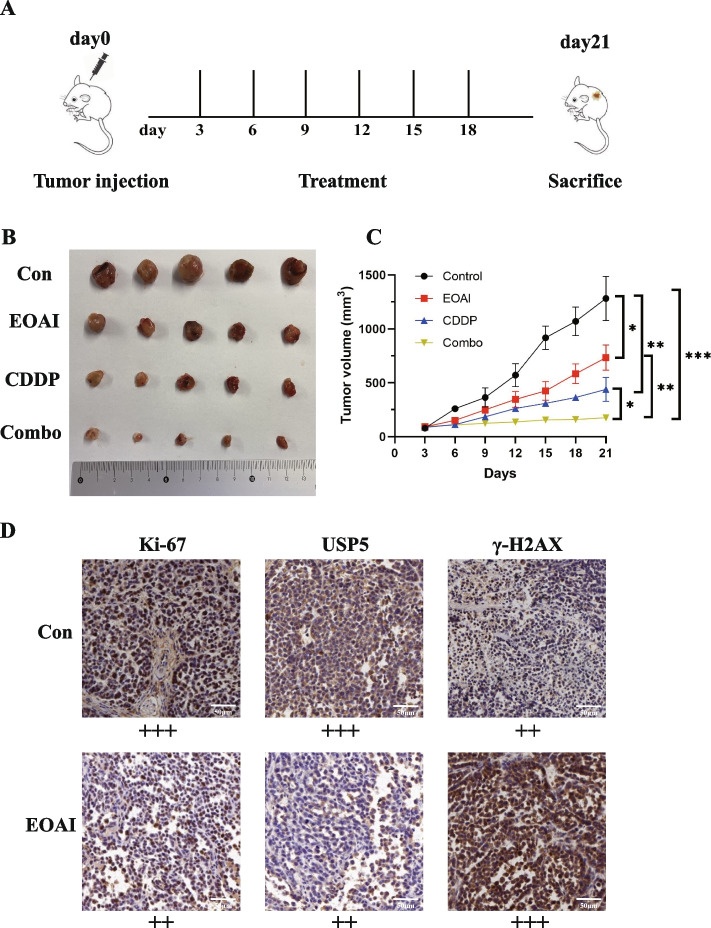
EOAI can enhance the effect of cisplatin anti-tumor. **A** Plant the mice Lewis lung cell (LLC) into the side abdominal skin of the C57 mice. When the tumor reaches 100 mm^3^, start EOAI and CDDP treatment. There are 5 animals in each group. Measure the volume of tumors and mice weight every 3 days. After 21 days, the mouse was executed, and the growth curve was drawn. **B** The mice were sacrificed on the 21st day of tumor formation, and the tumor was exfoliated and photographed. **C** Evaluate the difference between the size of mice tumors in different therapeutic groups through the t-test with GraphPad Prism8 software. **D** Immunohistochemical staining (IHC) of tumor tissue in mice. IHC was performed on tumor tissues of mice and classified according to the degree of staining, and reprehensive pictures were captured. Light yellow indicates weak positivity ( +), brownish yellow indicates moderate positivity (+ +), and brownish-black indicates strong positivity (+ + +)

We constructed a subcutaneous tumor model of lung cancer using C57 mice. The mice were divided into 4 groups of 5 each after 3 days of successful tumor inoculation and given drug treatment (Fig. 8A). The volume of tumors was measured and recorded every 3 days using vernier caliper. The mice were executed on day 21 after tumor formation, and the tumor tissues were removed and photographed for immunohistochemical (IHC) staining. The results showed that the tumor volume of mice in the EOAI group was significantly reduced compared to the control group. This reduction was more pronounced after combined treatment with CDDP (Fig. 8B). As evidenced by the size of tumors in mice, the two-drug combination group showed better efficacy than the single-drug group. Both single-drug and two-drug combinations showed anti-tumor effects (Fig. 8C). Immunohistochemical results showed that the expression of ki-67 and USP5 was higher in the control group than in the EOAI-treated mice. In contrast, the expression level of γ-H2AX was lower (Fig. 8D), which confirmed the results of our in vitro experiments.

## Discussion

The bottleneck of cancer treatment is the gradual development of drug resistance during treatment. Therefore, it is necessary to continuously explore the molecular mechanism of lung cancer progression and explore new drug therapeutic targets. There is much evidence that USP5 is involved in tumor progression, including promoting the process of tumor epithelial-mesenchymal transition (EMT), stabilizing cyclins to promote proliferation, activating inflammasome, etc. [23–25]. Therefore, USP5 is enormously appealing as a strategy for targeted tumor therapy. Our results suggested that USP5 is overexpressed in NSCLC tissues and cell lines, and this high expression is significantly correlated with tumor size and patient survival. This result provides a basis for the inhibition of USP5 to shrink the tumor for therapeutic purposes.

Based on the results of Kapuria V et al., drugs with DNBs inhibitory activity have shown attractive antitumor effects [26]. It is generally accepted that cancer cells exhibit more genomic instability and are more dependent on the DDR pathway to process endogenous and exogenous DNA damage [27]. When the DDR process is compromised, lethal DNA damage occurs, leading to cell growth arrest and apoptosis. Studies by Nakajima S et al. have shown that USP5 is involved in DNA repair and affects the efficiency of double-strand break (DSB) repair in homologous recombination by eliminating ubiquitin signaling at sites of DNA damage [28, 29]. These results demonstrate that tumor cells may have a greater need to maintain USP5 activity and, thus, DNA stability than normal cells. Consistent with these reports, our results showed that inhibition of USP5 cells showed a longer tail in the comet assay. The immunofluorescence results showed that inhibition of USP5 resulted in more γ-H2AX-positive cells and a significant upregulation of γ-H2AX after inhibition of USP5 was detected at the protein level, indicating significant DNA damage in NSCLC cells.

Ubiquitination is involved in the modification, degradation and functional regulation of proteins and has been extensively studied in the context of DNA damage response (DDR). Different ubiquitination pathways have various molecular structural features and biological functions, including ubiquitin chains that play essential functions in the DDR process [30]. In recent years, it has become apparent that altering such ubiquitination by DUBs plays a crucial role in regulating DNA damage-related events and the activities they control [31]. EOAI, an inhibitor of USP5 deubiquitinating enzymes, showed a dose-dependent tumor growth inhibition in our results. Mechanistically, EOAI can trigger DNA damage in NSCLC cells, causing a significant upregulation of the appearance of γ-H2AX expression. At the molecular level, we found that EOAI induced DNA damage, activated p53, and effectively inhibited NSCLC cell growth through cell cycle arrest and apoptosis.

In DNA damage, p53 is considered a decision-making transcription factor that selectively activates genes as part of specific gene expression programs to determine cellular outcomes [32]. Like most post-translational modifications, the ubiquitination of p53 can be reversed by the counteracting action of deubiquitinases. Some USP family members have been shown to regulate the p53 pathway [33, 34]. Studies have shown that multiple genes involved in cell cycle regulation are repressed in a p53-dependent manner [35]. The p53 tumor suppressor protein has a vital role in protecting genome integrity. In unstressed cells, p53 is maintained at low levels through the ubiquitin–proteasome pathway, and activated p53 transcriptionally regulates multiple biological processes, including DNA damage repair, cell cycle arrest, apoptosis, and autophagy. Das S et al. showed that the p53 target gene encoding zinc finger protein 16 binds to p53 and increases its binding and transactivation to the cell cycle arrest genes CDKN1A and SFN, thereby causing cell cycle arrest [36]. Our study found that after inhibition of USP5, NSCLC cells were arrested in the G2/M phase, and further protein level analysis found that cells were mainly arrested in the G2 phase. Following the addition of the p53 inhibitor PFT, G2/M arrest was reduced, and there was also a callback in cyclin levels. The above results suggest that the cycle arrest caused by EOAI may be caused by the activation of p53 by DNA damage.

Studies have shown that P21 can increase the levels of pro-apoptotic proteins such as Bax, and change mitochondrial permeability, thereby playing a pro-apoptotic role [37]. Previous studies have reported increased apoptosis in tumor cells, and the upregulation of the apoptotic protein Noxa depended on the activation of p53 [17]. Similarly, after inhibition of USP5, we found that NSCLC cell apoptosis was increased, while the expression of pro-apoptotic proteins was up-regulated, and anti-apoptotic proteins were down-regulated. Inactivation of p53 reduced EOAI-induced apoptosis and the expression of the apoptotic protein Noxa after adding the p53 inhibitor PFT. Furthermore, we demonstrated that blocking endogenous Noxa induction can lead to the inhibition of NSCLC cell apoptosis. This is consistent with the results of Zhendong Yu et al., which showed that cell DNA damage could promote apoptosis of tumor cells through the p53-PUMa /NOXA/ BCl2-Bax pathway, and kill cancer cells by inducing cell cycle arrest through the p53-P21 pathway [38].

The reports of DUBs stably participating in autophagy-related proteins by deubiquitination and their involvement in autophagy are not isolated cases. The study by Xiao W et al. found that E2F4 (E2F transcription factor 4) directly regulates the transcription of autophagy-related proteins (autophagy-related 2A and unc-51 like autophagy activating kinase 2). At the same time, USP2 stabilizes the E2F4 protein through deubiquitination, reducing its transactivation in tumor cells and thereby inhibiting its protective autophagy [39]. Similarly, Cai B et al. demonstrated that USP5 could promote autophagic degradation [25]. Our results also prove that inhibition of USP5 can promote autophagy in NSCLC cells, and this promotion effect is inhibited after the inhibition of p53. Mechanistically, after toxic gene stress, p53 activates AMPK while inhibiting mTOR, translation and ribosome biosynthesis are inhibited, and autophagy is activated [40]. Adenosine monophosphate-activated protein kinase (AMPK) was demonstrated by Egan DF et al. in 2011 to induce autophagy in mammalian cells by activating ULK1 and ULK2 [41]. Our study confirmed that EOAI triggered autophagy in cells, and the addition of autophagy inhibitors enhances EOAI-induced apoptosis in NSCLC cells. In addition, the activity of AMPK was down-regulated after adding PFT, while EOAI-induced autophagy in NSCLC cells was also inhibited.

After a series of in vivo and in vitro studies, EOAI has been shown to have a promising antitumor effect in various tumors [42–44]. In vivo experiments, EOAI has been shown to reduce tumor volume and, when combined with cisplatin, to have a better antitumor effect than a single agent. Inactivation of USP5 by EOAI can effectively inhibit the growth of NSCLC cells in vitro or in mice. These results suggest targeting USP5 as a potential anti-NSCLC therapeutic strategy.

Collectively, we observed significant DNA damage in NSCLC treatment with the USP5 inhibitor EOAI, as well as cell cycle arrest, apoptosis, and autophagy induced by p53 transcription factor activation. These results enrich the epigenetic relationship of NSCLC, suggesting that EOAI may be a new therapeutic agent targeted to kill NSCLC cells, providing theoretical support for EOAI to inhibit tumor growth in vivo. In addition, we provided information on the expression levels of USP5 in NSCLC tissues and cell lines. From the perspective of expression, the high expression of USP5 indicates a poor prognosis, suggesting that USP5 can not only be used as a therapeutic target of NSCLC but also may have the potential as a diagnostic marker of NSCLC.

## Conclusions

Our findings create new insights into the development of DUB inhibitors and may add to the limited repertoire of current NSCLC treatments. Through inhibition of USP5, p53 and rescue experiments, it was shown that USP5 activates the transcription of p53 by causing DNA damage, thereby causing an increase in apoptosis, autophagy and cycle arrest. In vivo experiments showed that combined treatment with chemotherapy drugs and USP5 inhibitors fully suppressed tumor growth. By revealing the stringent regulation of DNA damage mediated by various DUBs in NSCLC, our study might yield novel diagnostic and therapeutic targets in the future.

## Supplementary Information

**Additional file 1.**

**Additional file 2.**

## Data Availability

All datasets and materials generated for this study are included in the manuscript/supplementary files.
