# Supplementary material for: EOAI, a ubiquitin-specific peptidase 5 inhibitor, prevents non-small cell lung cancer progression by inducing DNA damage
Source: BMC Cancer. 2023 Jan 7;23:28. doi: 10.1186/s12885-023-10506-0 (PMC9826599; doi:10.1186/s12885-023-10506-0)
Supplement: Supplementary file 1 — Additional file 1. [file 12885_2023_10506_MOESM1_ESM.pdf]

Figure1-D

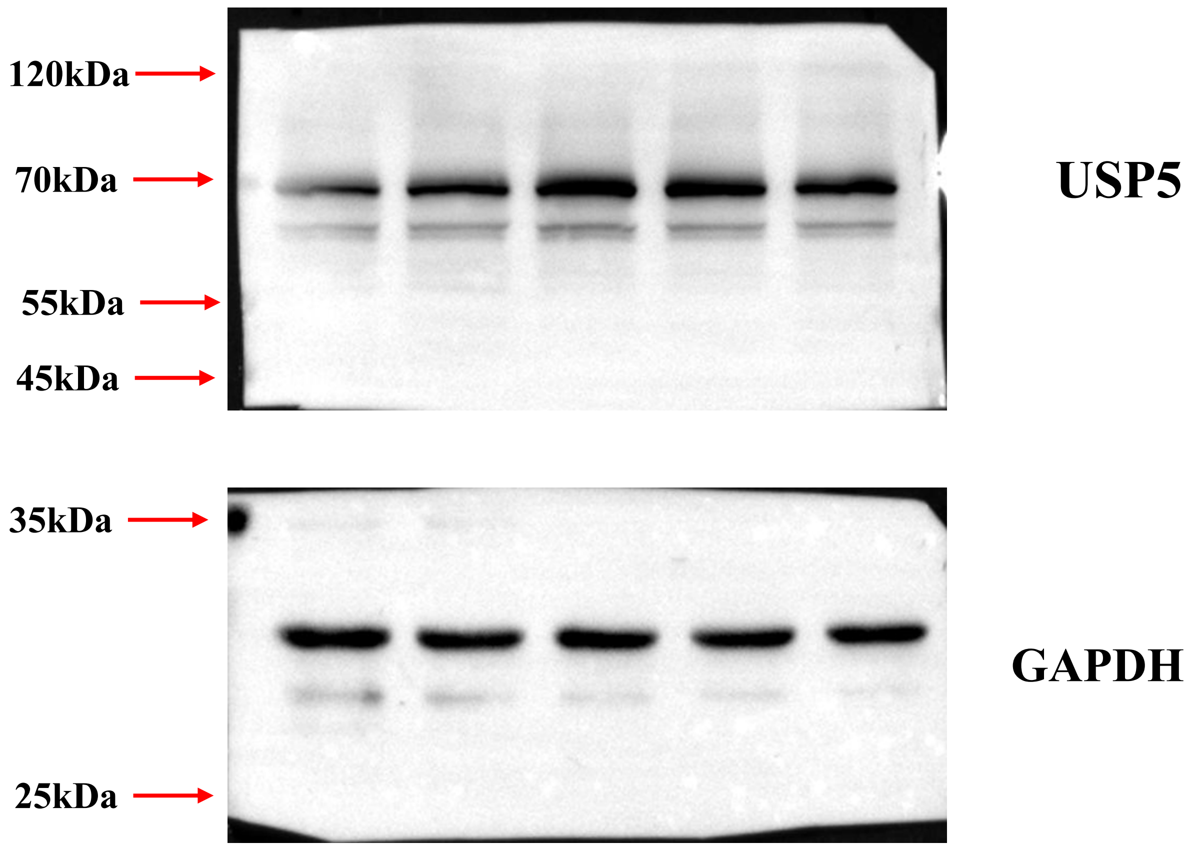

Figure2-C

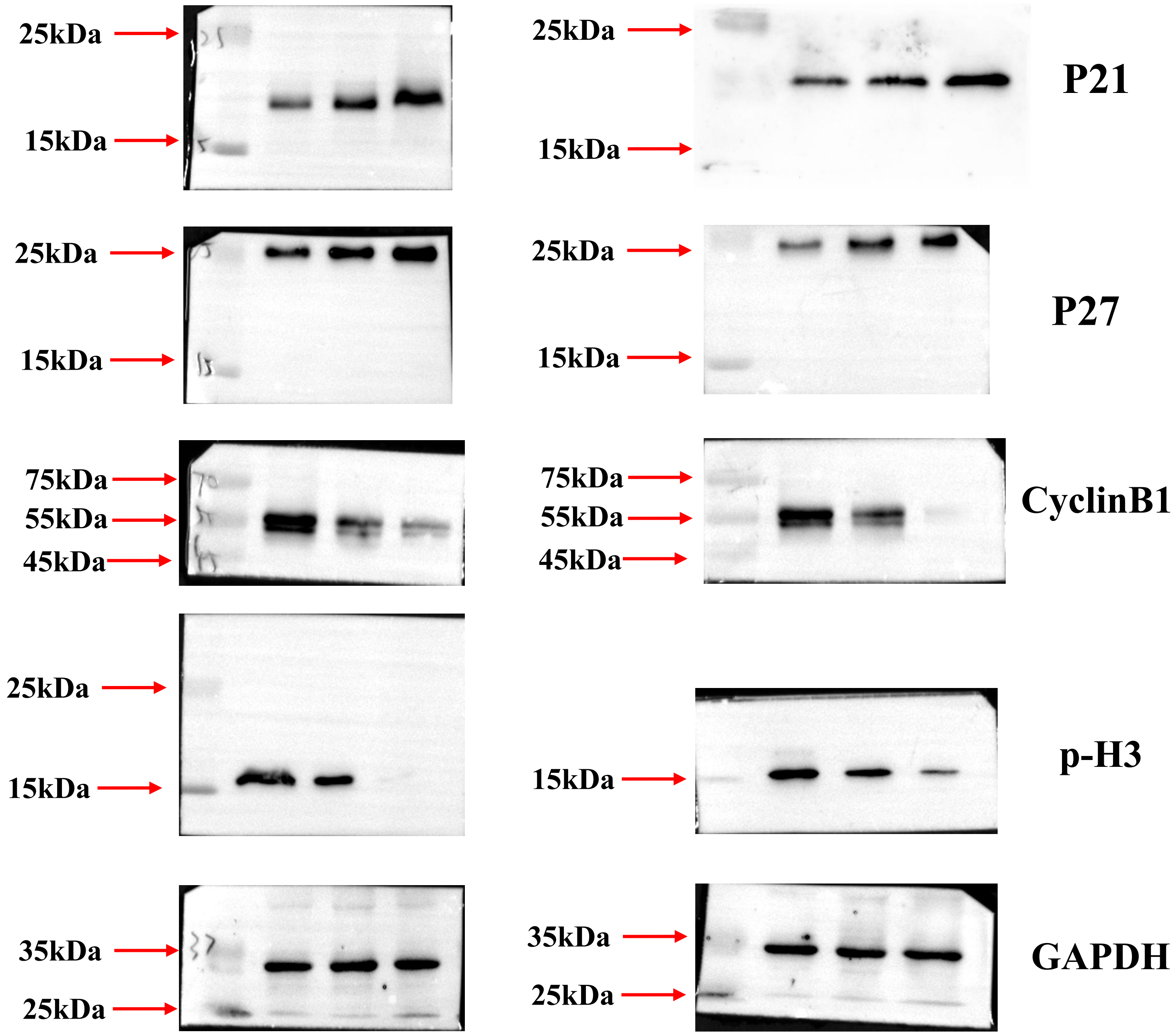

25kDa
15kDa
25kDa
15kDa
P21
25kDa
15kDa
25kDa
15kDa
P27
75kDa
55kDa
45kDa
75kDa
55kDa
45kDa
CyclinB1
25kDa
15kDa
15kDa
p-H3
35kDa
25kDa
35kDa
25kDa
GAPDH

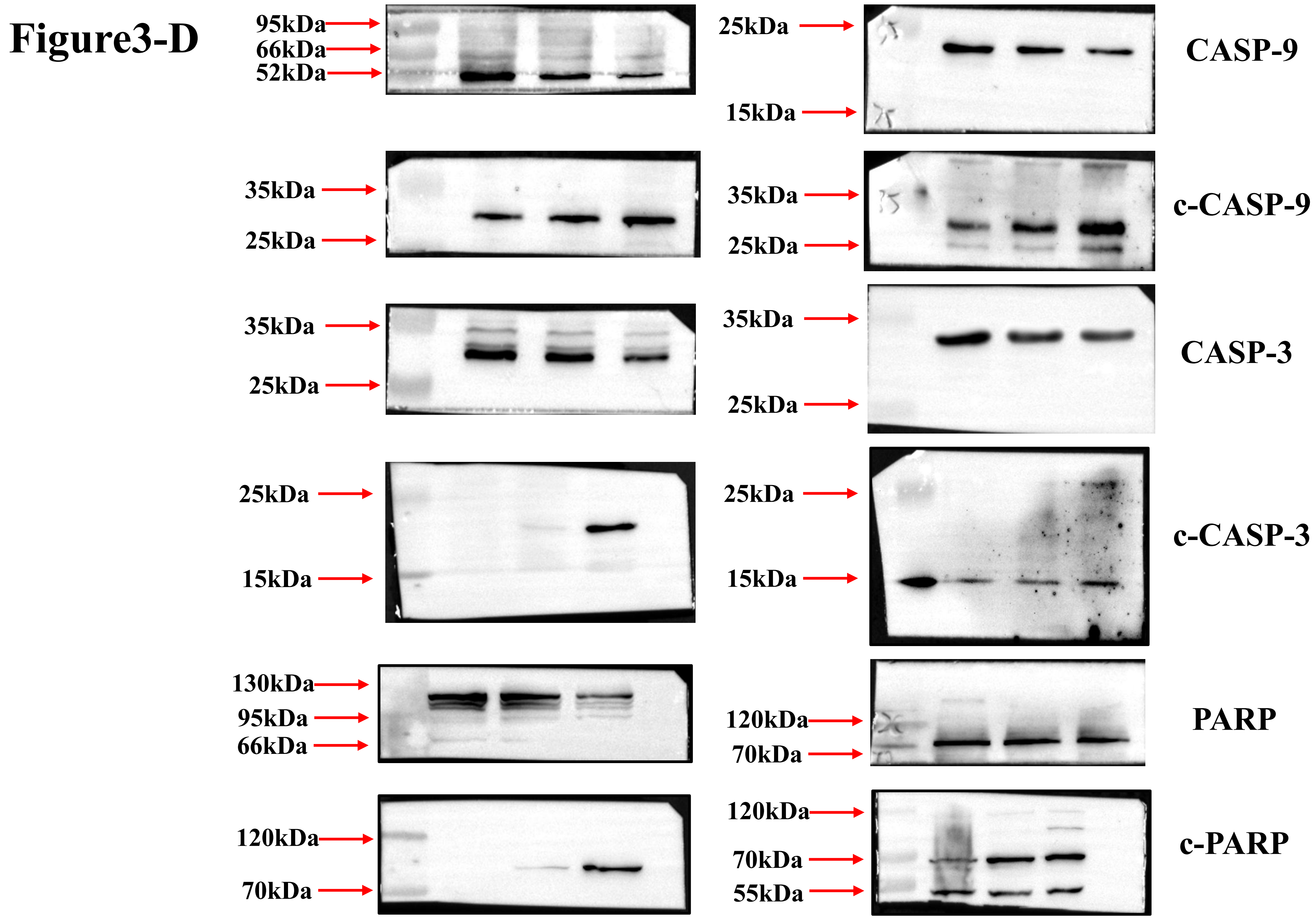

Figure3-D
95kDa
66kDa
52kDa
25kDa
15kDa
CASP-9
35kDa
25kDa
35kDa
25kDa
c-CASP-9
35kDa
25kDa
35kDa
25kDa
CASP-3
25kDa
15kDa
25kDa
15kDa
c-CASP-3
130kDa
95kDa
66kDa
120kDa
70kDa
PARP
120kDa
70kDa
120kDa
70kDa
55kDa
c-PARP

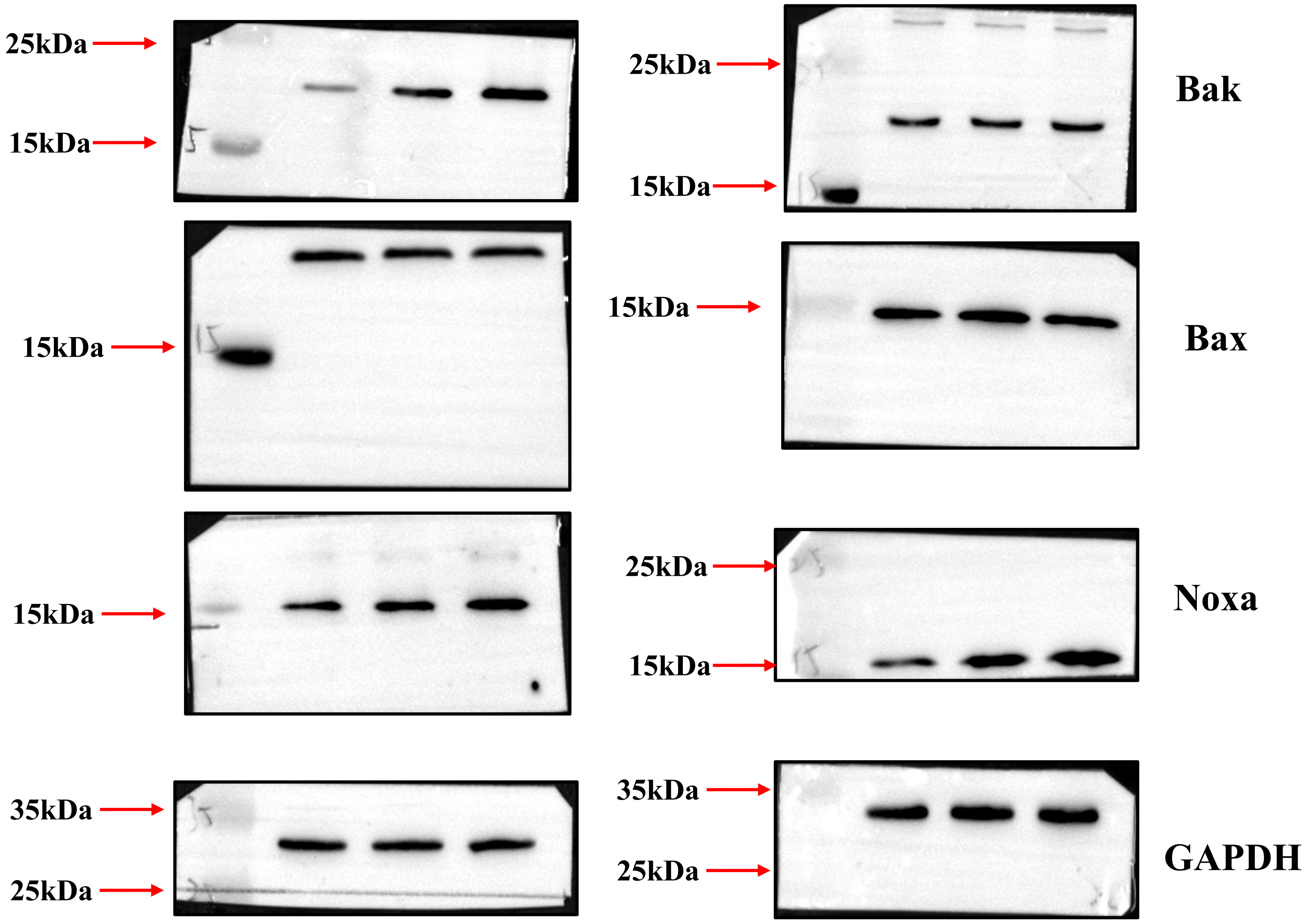
25kDa
15kDa
25kDa
15kDa
Bak
15kDa
15kDa
Bax
15kDa
25kDa
15kDa
Noxa
35kDa
25kDa
35kDa
25kDa
GAPDH

Figure3-E

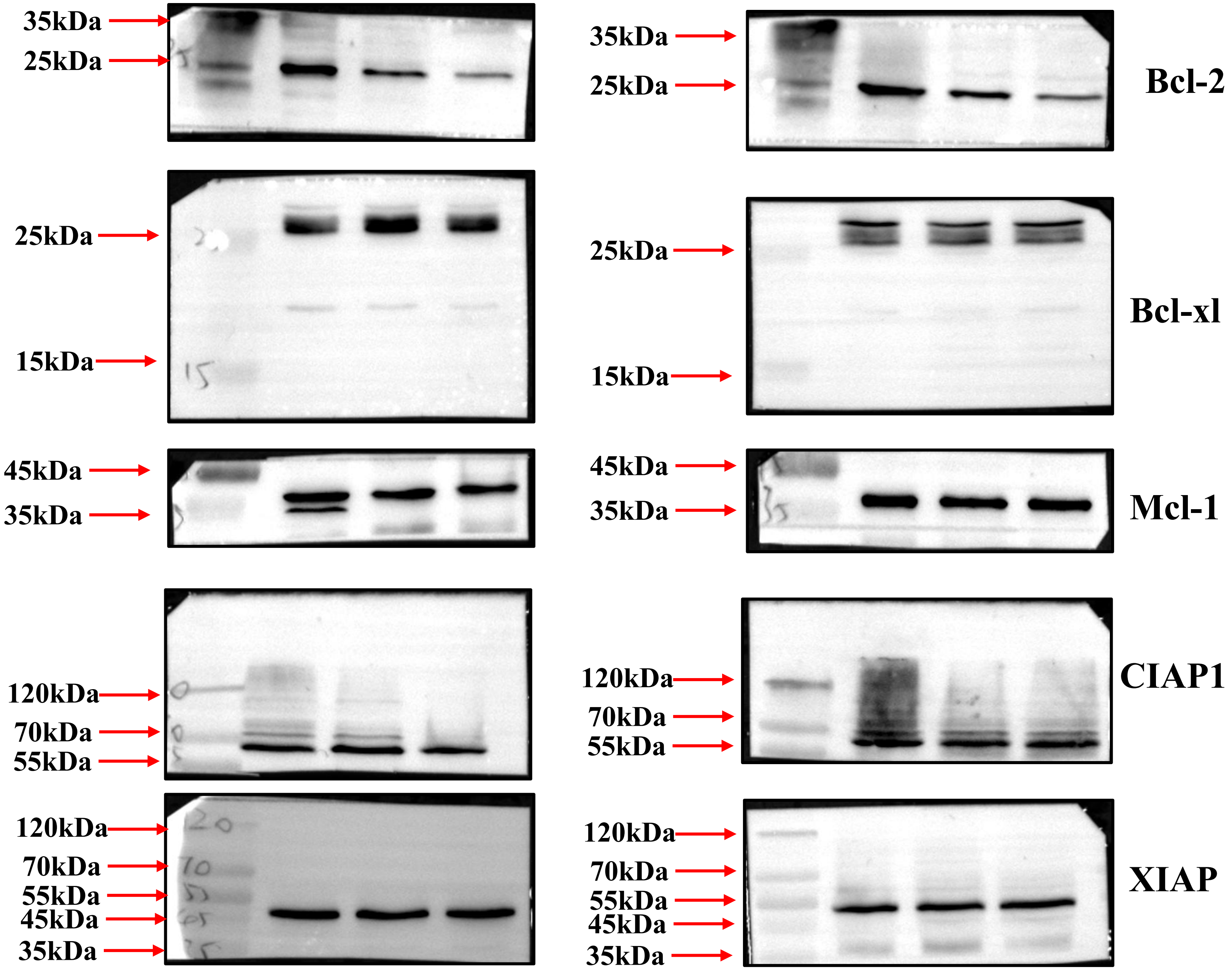
35kDa
25kDa
Bcl-2
25kDa
15kDa
Bcl-xl
45kDa
35kDa
Mcl-1
120kDa
70kDa
55kDa
CIAP1
120kDa
70kDa
55kDa
45kDa
35kDa
XIAP

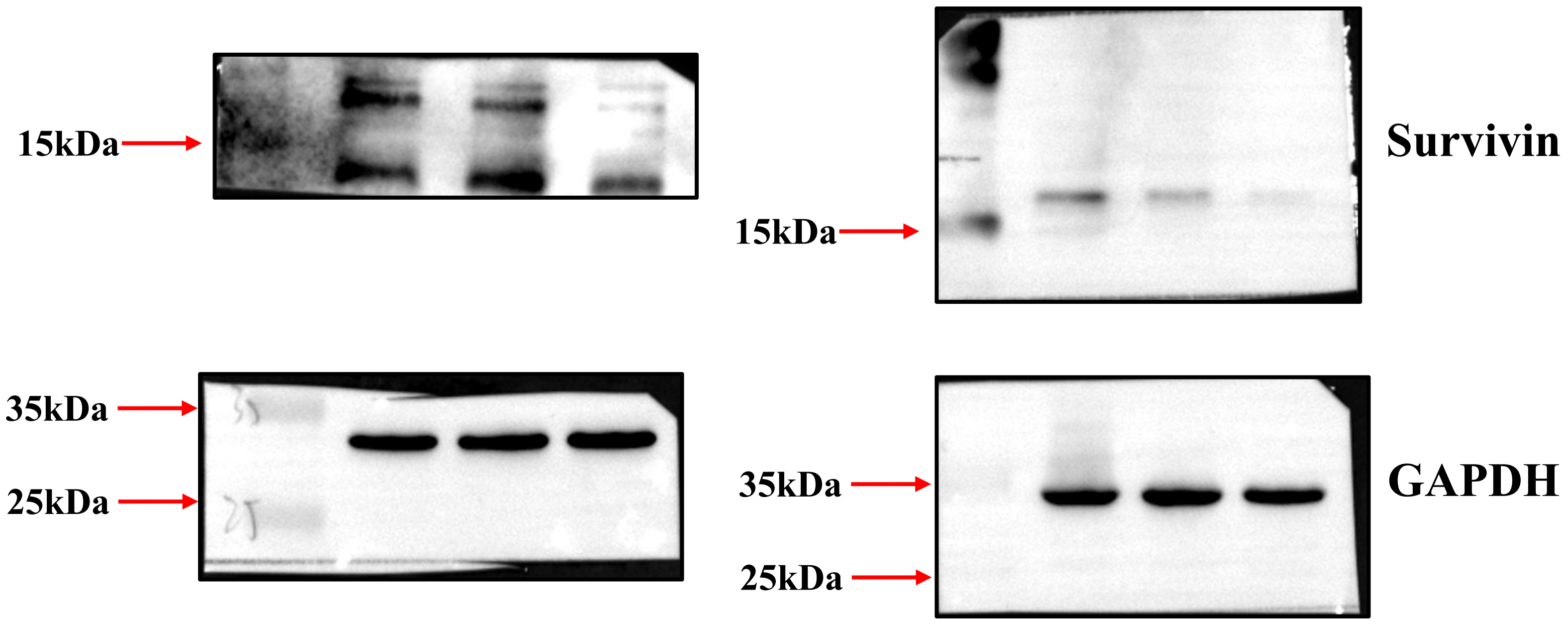
15kDa
15kDa
Survivin
35kDa
25kDa
35kDa
25kDa
GAPDH

Figure4-B

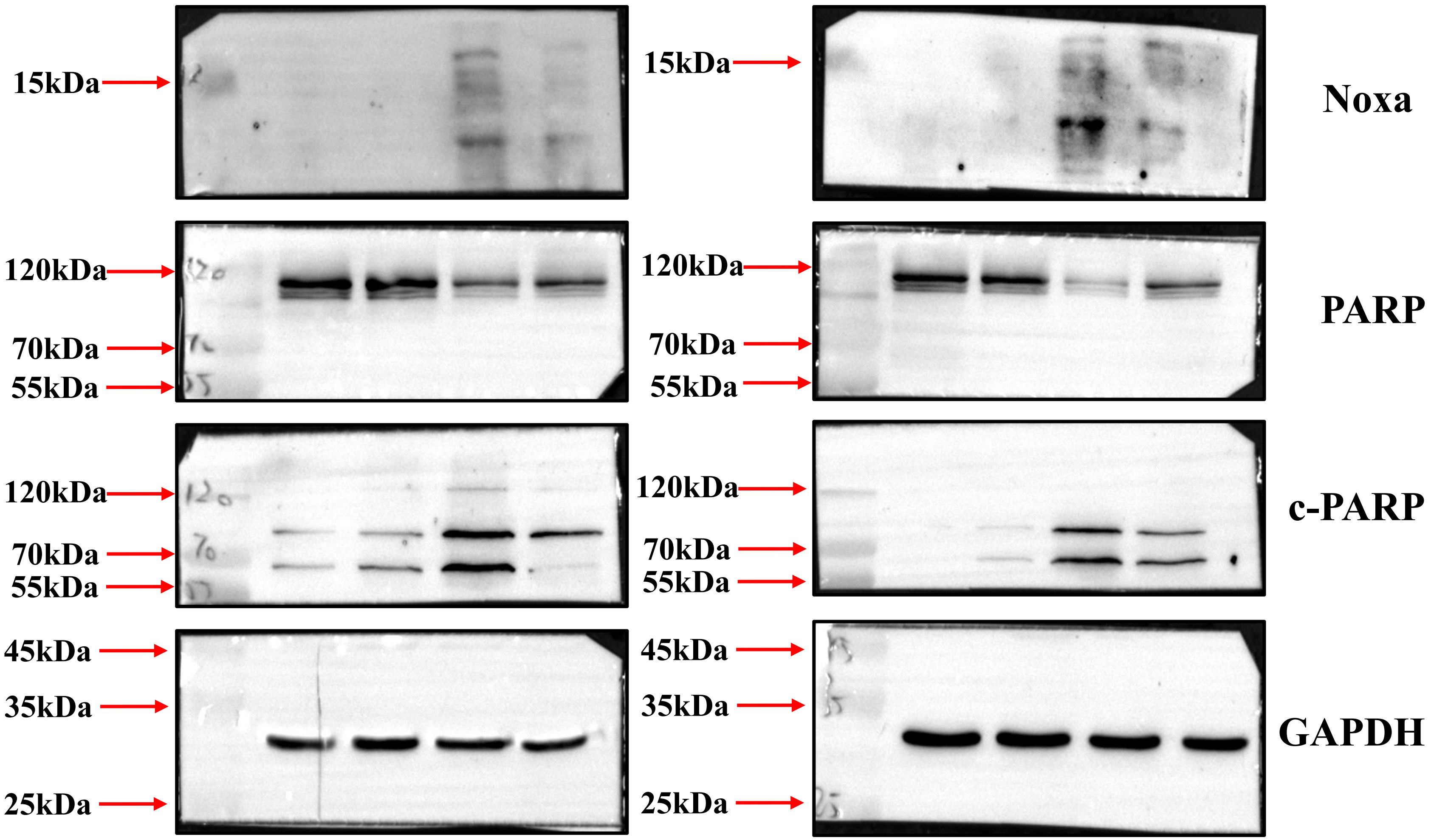
15kDa
15kDa
Noxa
120kDa
70kDa
55kDa
120kDa
70kDa
55kDa
PARP
120kDa
70kDa
55kDa
120kDa
70kDa
55kDa
c-PARP
45kDa
35kDa
25kDa
45kDa
35kDa
25kDa
GAPDH

Figure4-C

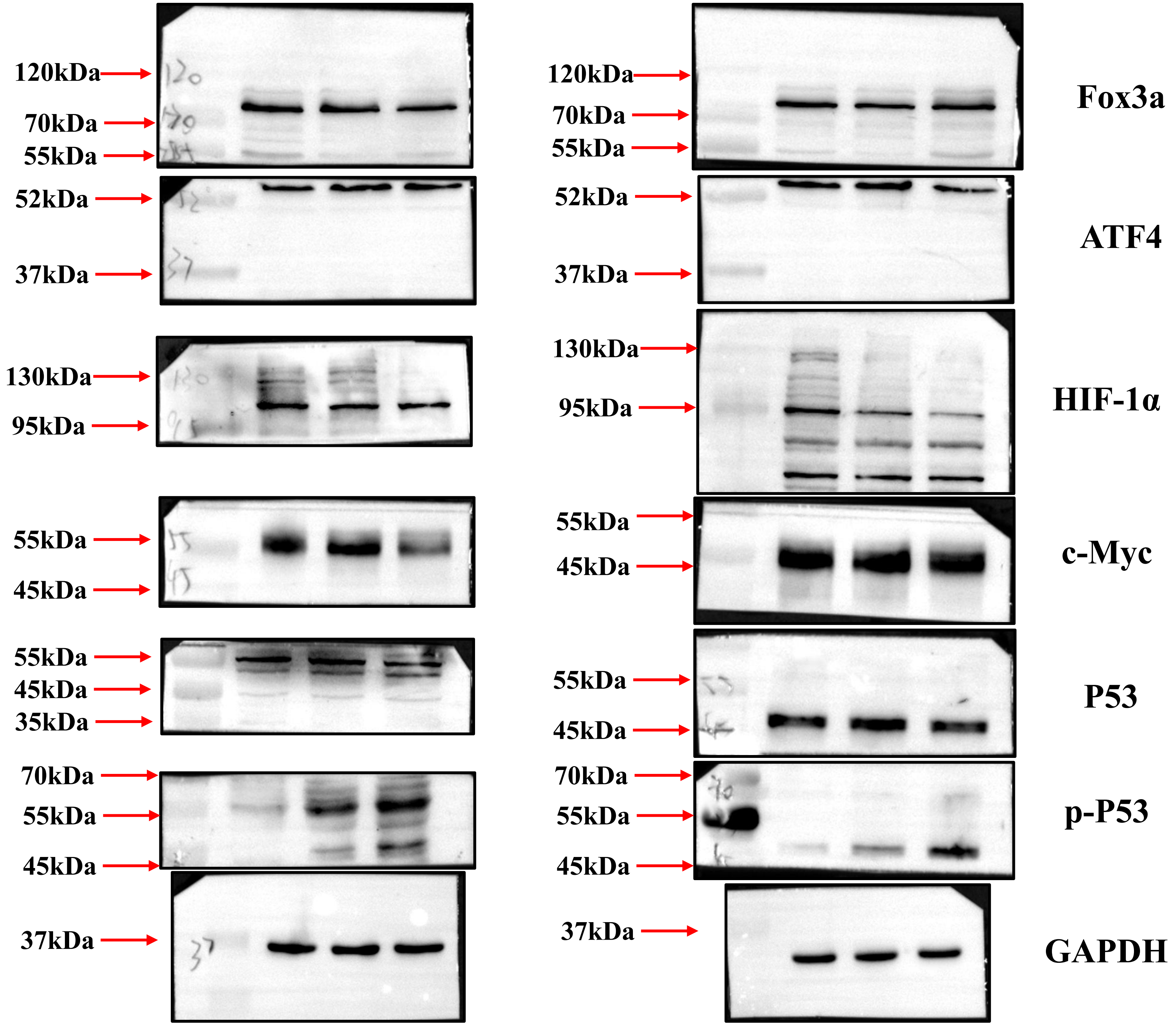
120kDa
70kDa
55kDa
52kDa
37kDa
130kDa
95kDa
55kDa
45kDa
55kDa
45kDa
35kDa
70kDa
55kDa
45kDa
37kDa
120kDa
70kDa
55kDa
Fox3a
52kDa
37kDa
ATF4
130kDa
95kDa
HIF-1α
55kDa
45kDa
c-Myc
55kDa
45kDa
P53
70kDa
55kDa
45kDa
p-P53
37kDa
GAPDH

Figure4-E

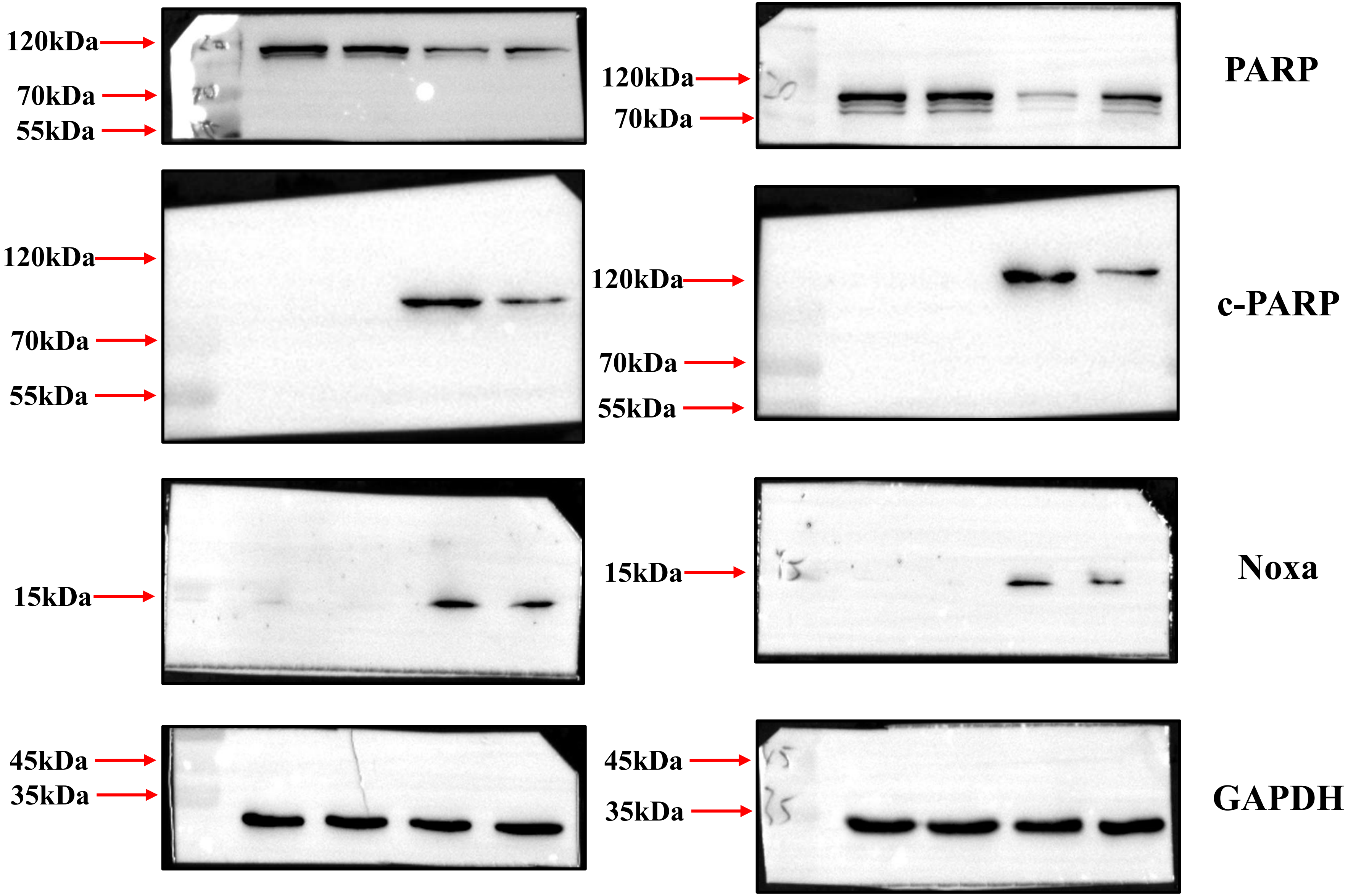

120kDa
70kDa
55kDa
120kDa
70kDa
PARP
120kDa
70kDa
55kDa
120kDa
70kDa
55kDa
c-PARP
15kDa
15kDa
Noxa
45kDa
35kDa
45kDa
35kDa
GAPDH

Figure5-B

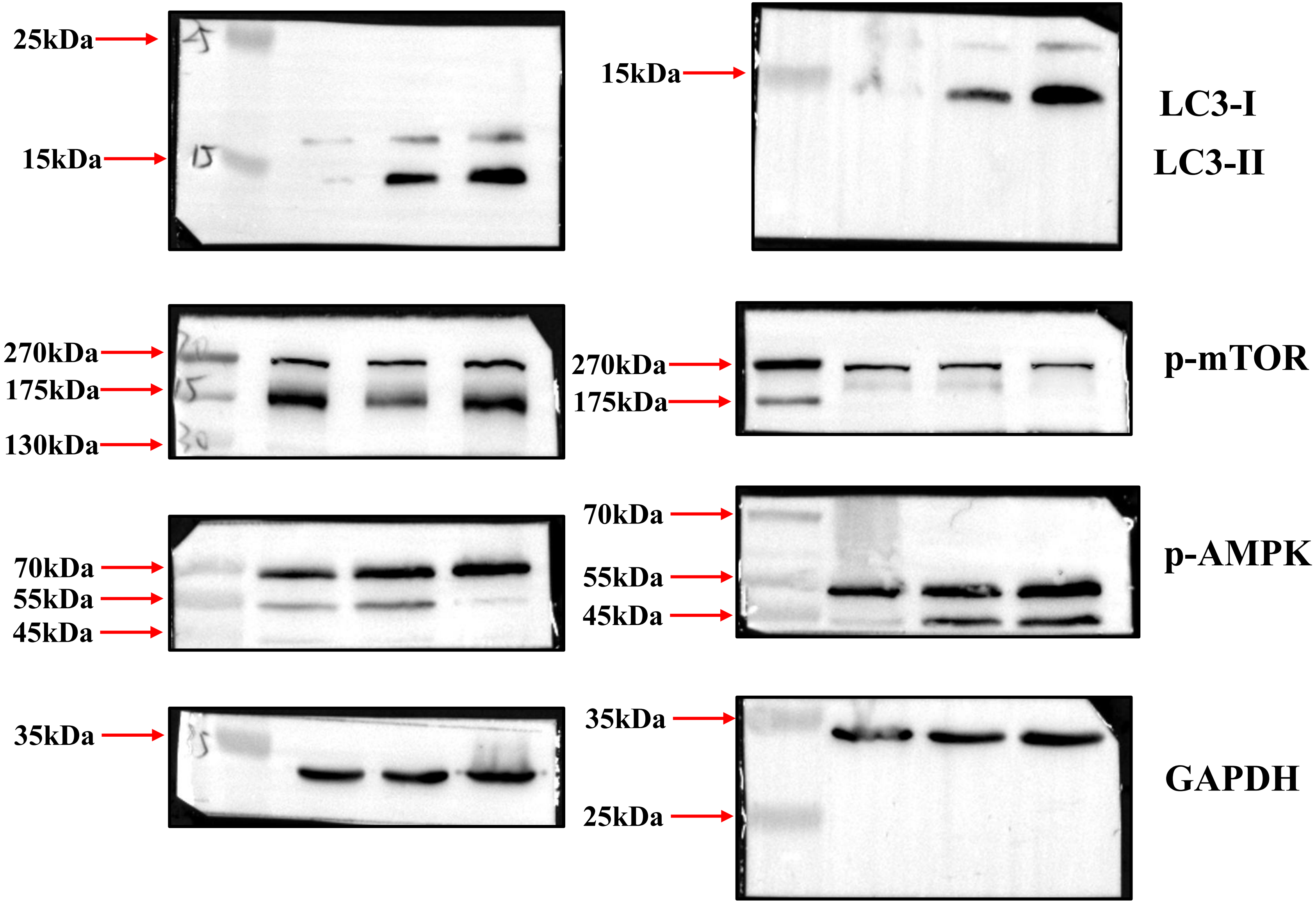
25kDa
15kDa
15kDa
LC3-I
LC3-II
270kDa
175kDa
130kDa
270kDa
175kDa
p-mTOR
70kDa
55kDa
45kDa
70kDa
55kDa
45kDa
p-AMPK
35kDa
35kDa
25kDa
GAPDH

Figure5-F

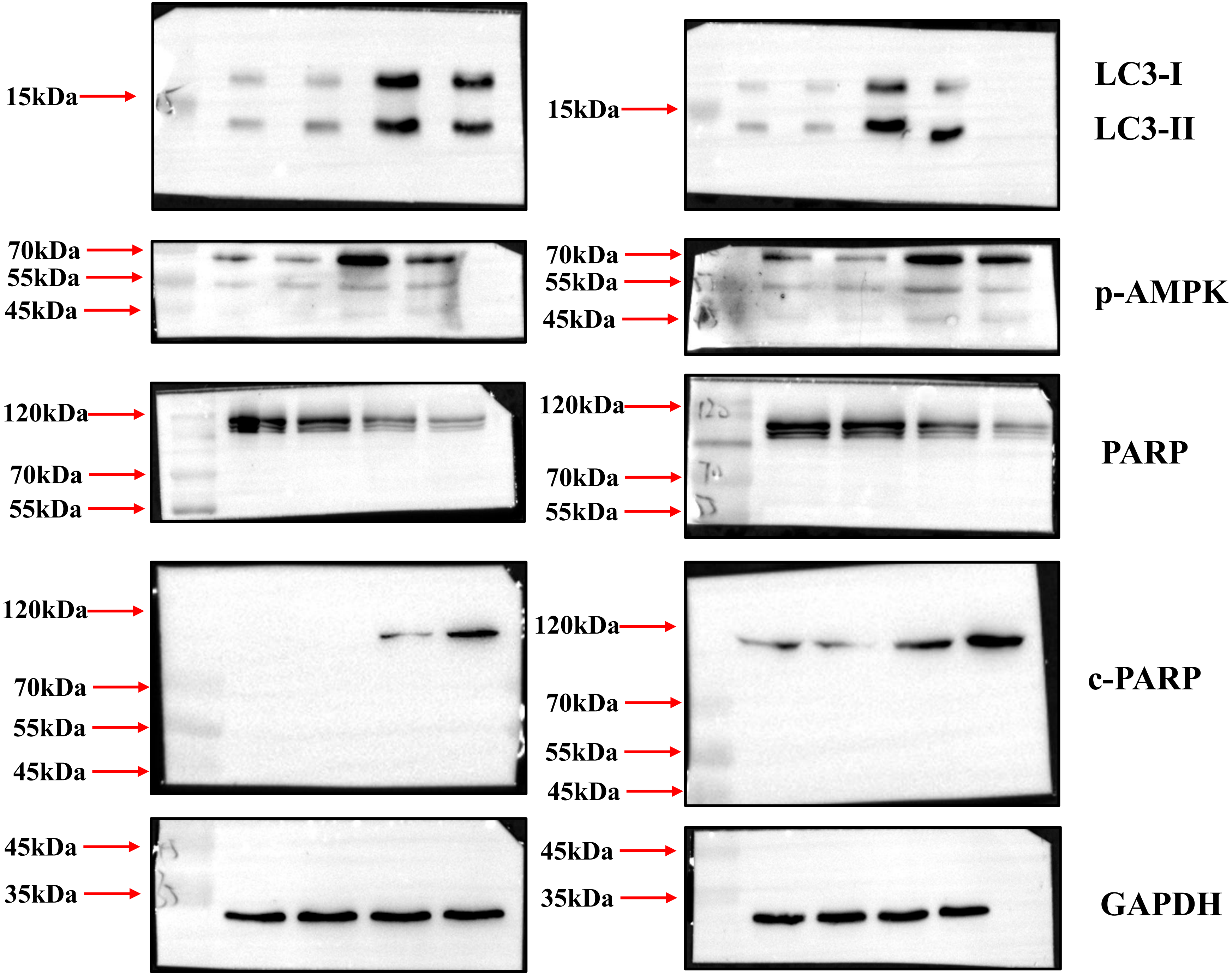

15kDa
15kDa
LC3-I
LC3-II
70kDa
55kDa
45kDa
70kDa
55kDa
45kDa
p-AMPK
120kDa
70kDa
55kDa
120kDa
70kDa
55kDa
PARP
120kDa
70kDa
55kDa
45kDa
120kDa
70kDa
55kDa
45kDa
c-PARP
45kDa
35kDa
45kDa
35kDa
GAPDH

Figure6-C

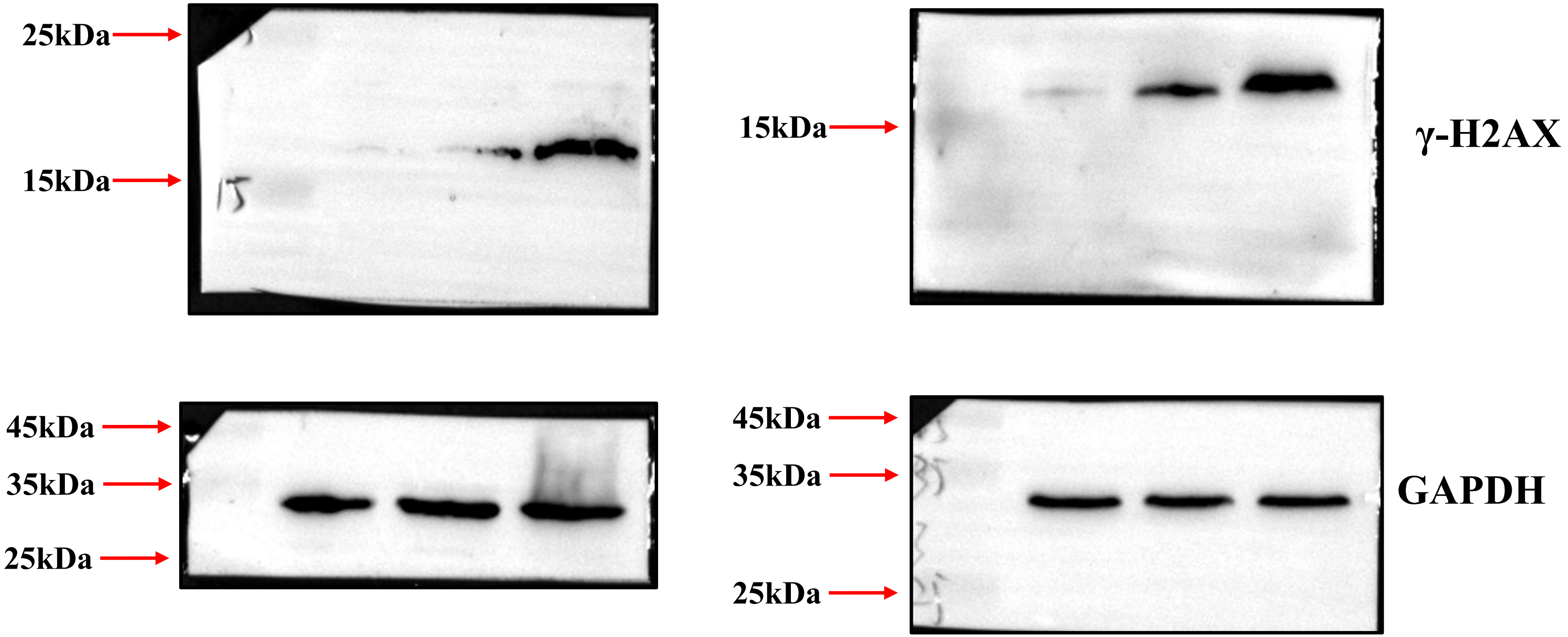
25kDa
15kDa
15kDa
γ-H2AX
45kDa
35kDa
25kDa
45kDa
35kDa
25kDa
GAPDH

Figure6-D

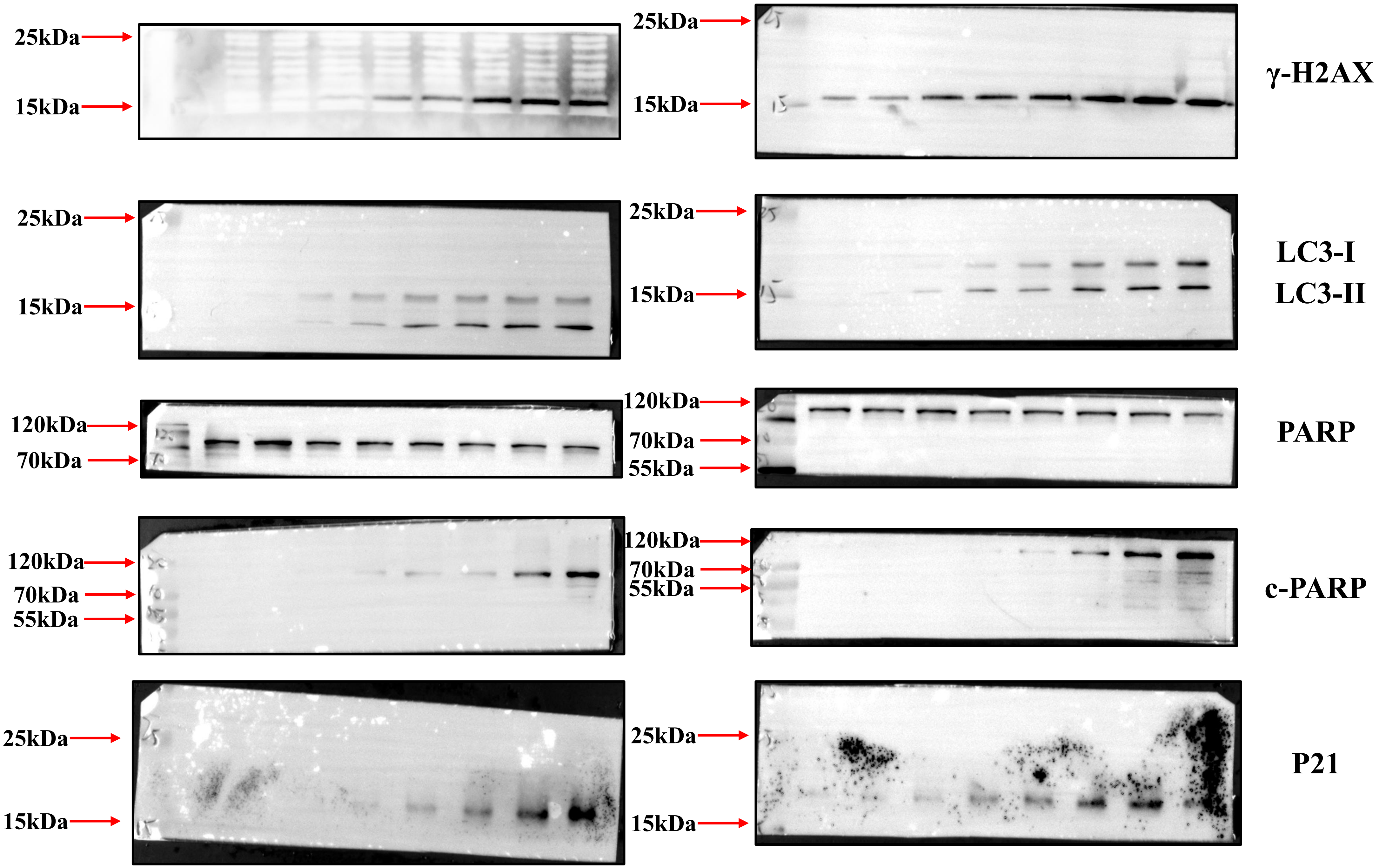
25kDa
15kDa
25kDa
15kDa
γ-H2AX
25kDa
15kDa
25kDa
15kDa
LC3-I
LC3-II
120kDa
70kDa
120kDa
70kDa
55kDa
PARP
120kDa
70kDa
55kDa
120kDa
70kDa
55kDa
c-PARP
25kDa
15kDa
25kDa
15kDa
P21

Figure6-D

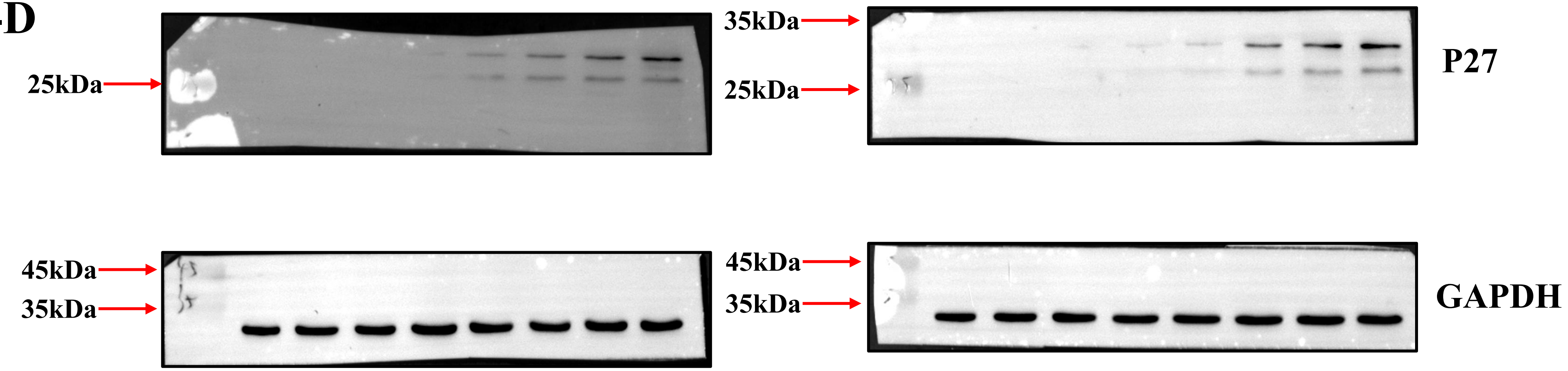

Figure7-C

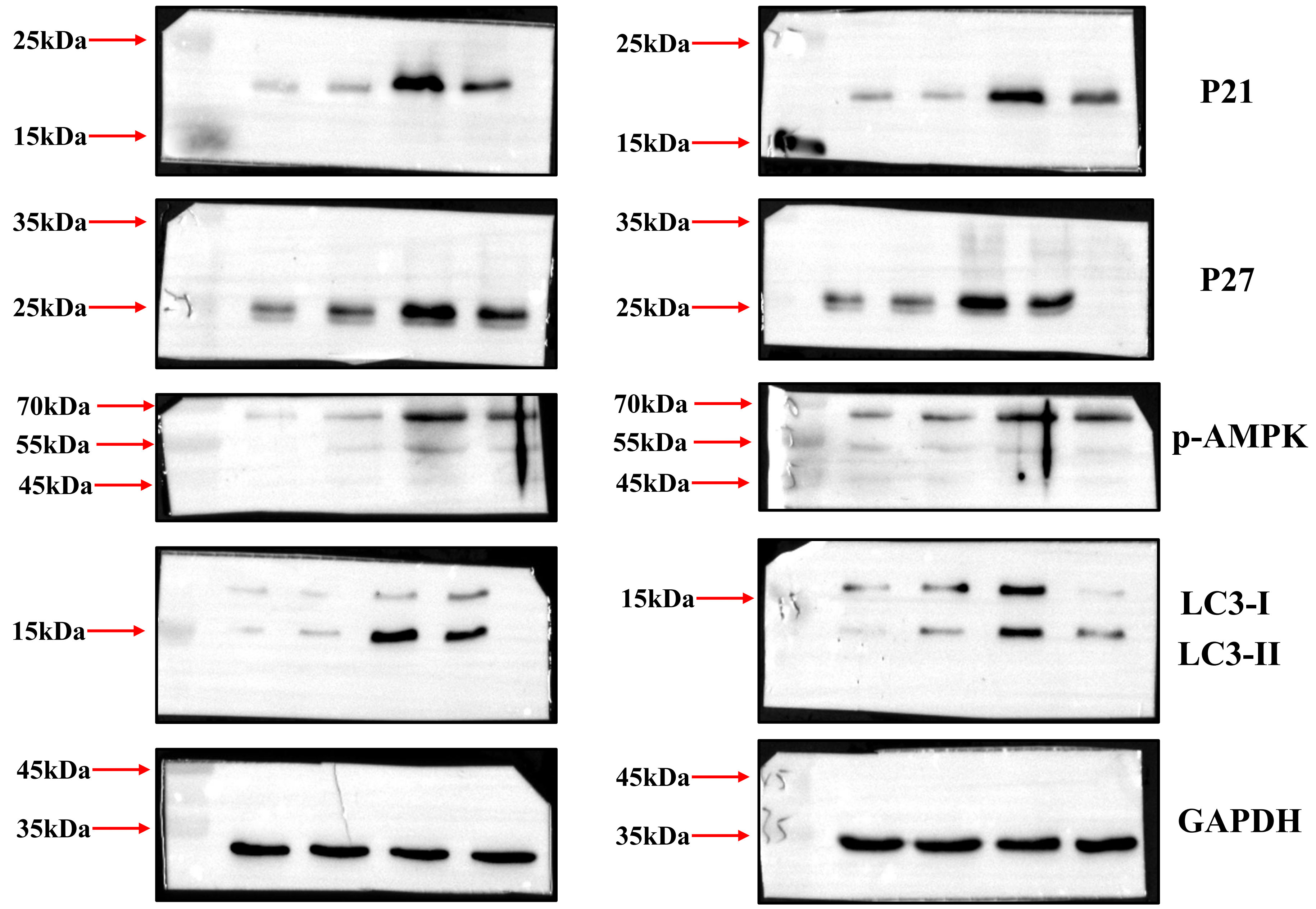
25kDa
15kDa
25kDa
15kDa
P21
35kDa
25kDa
35kDa
25kDa
P27
70kDa
55kDa
45kDa
70kDa
55kDa
45kDa
p-AMPK
15kDa
15kDa
LC3-I
LC3-II
45kDa
35kDa
45kDa
35kDa
GAPDH
