# Supplementary material for: EOAI, a ubiquitin-specific peptidase 5 inhibitor, prevents non-small cell lung cancer progression by inducing DNA damage
Source: BMC Cancer. 2023 Jan 7;23:28. doi: 10.1186/s12885-023-10506-0 (PMC9826599; doi:10.1186/s12885-023-10506-0)

A549 DMSO Gate1

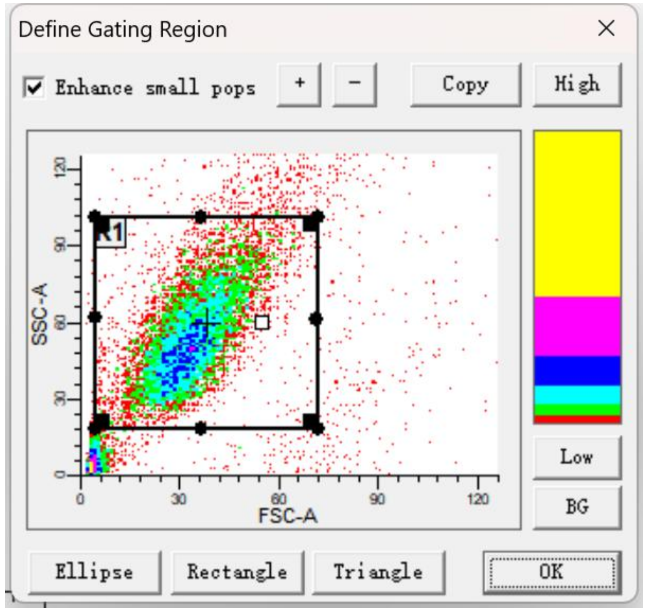

A549 DMSO Gate2

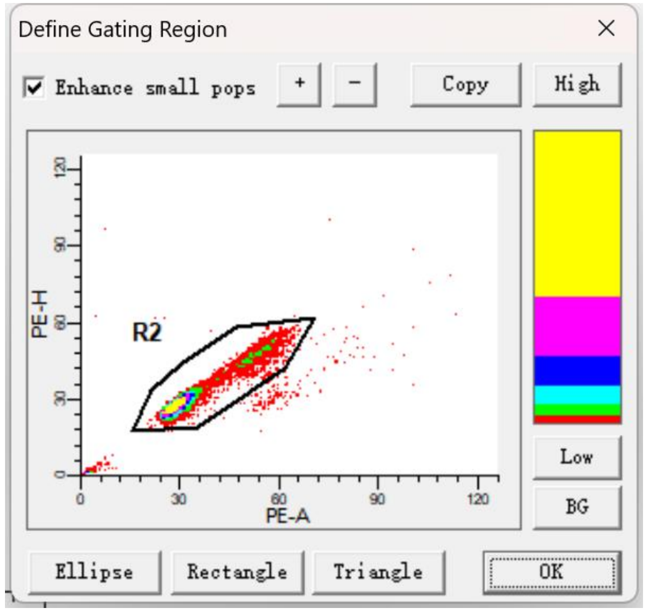

A549 2μM Gate1

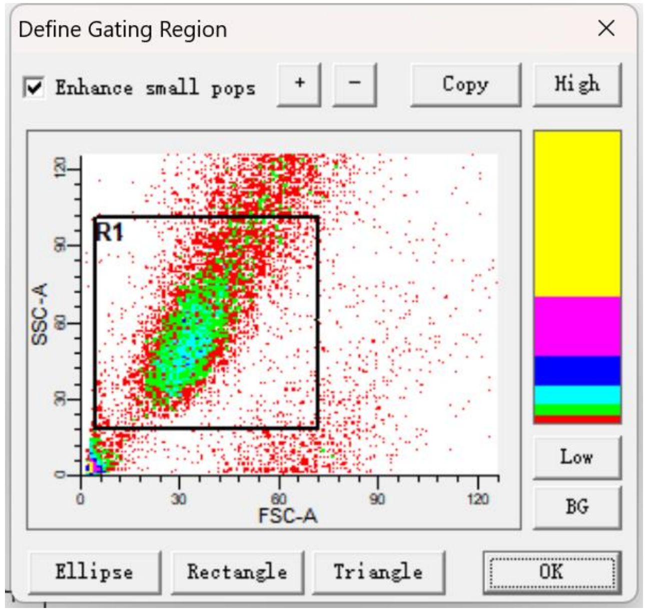

A549 2μM Gate2

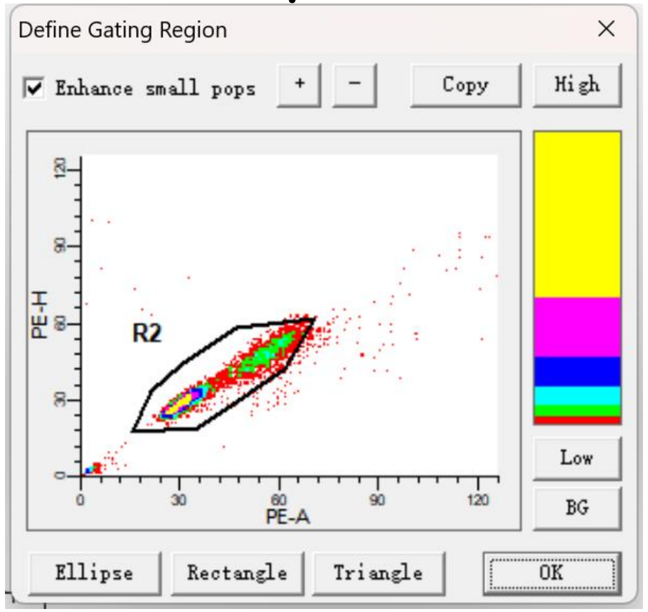

A549 4μM Gate1

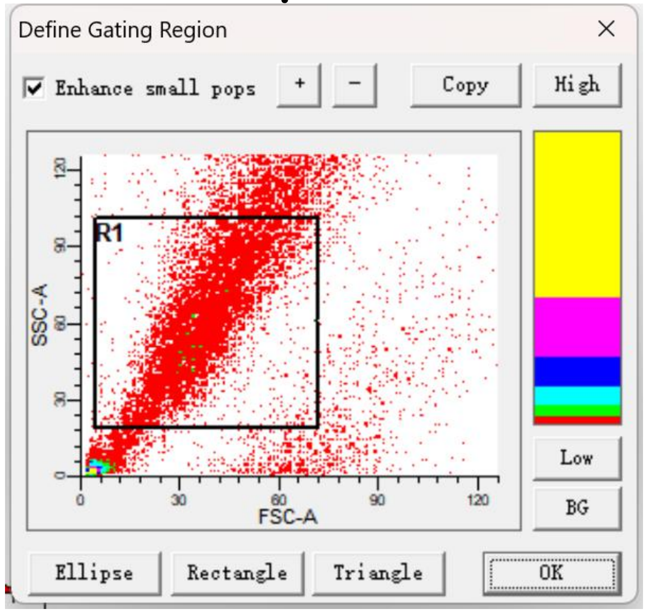

A549 4μM Gate2

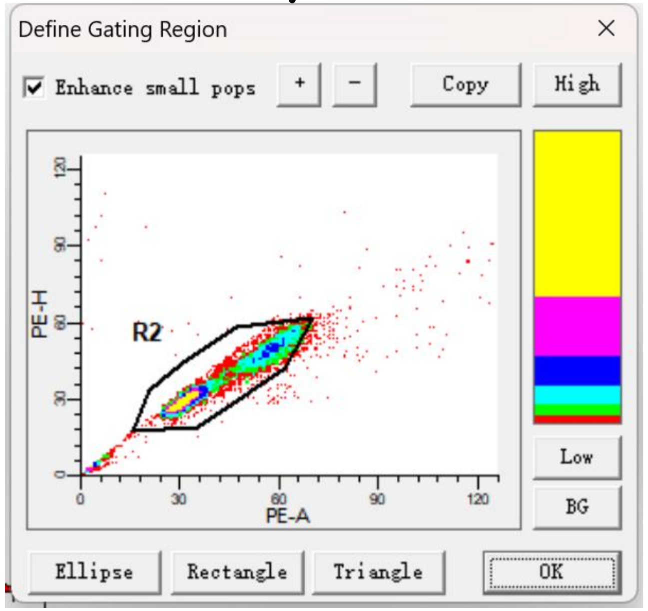

H460 DMSO Gate1

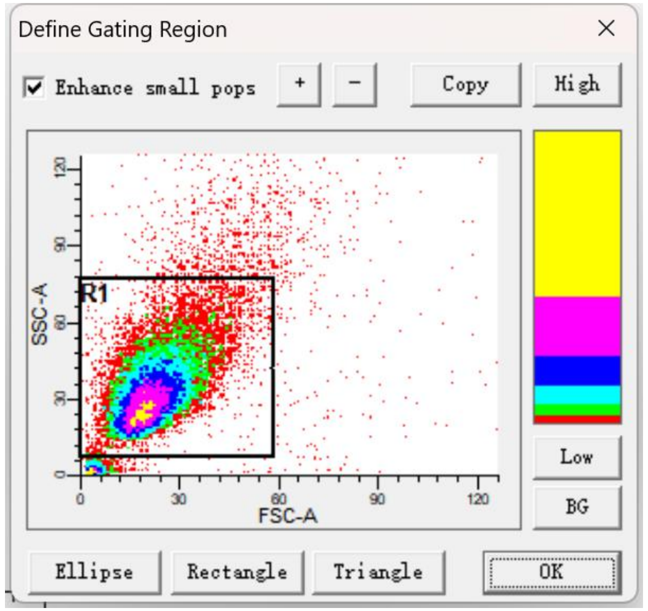

H460 DMSO Gate2

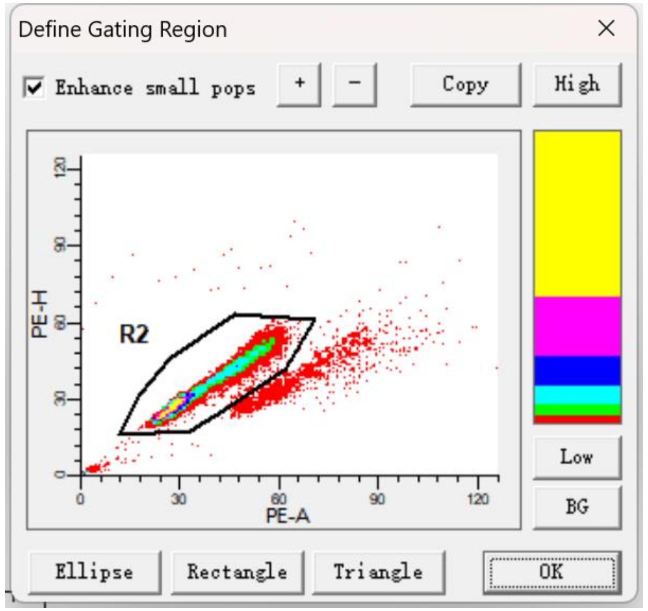

H460 2μM Gate1

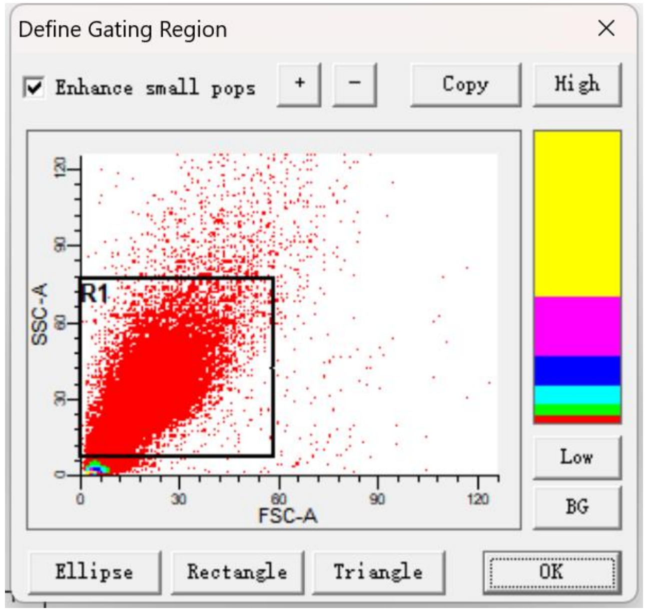

H460 2μM Gate2

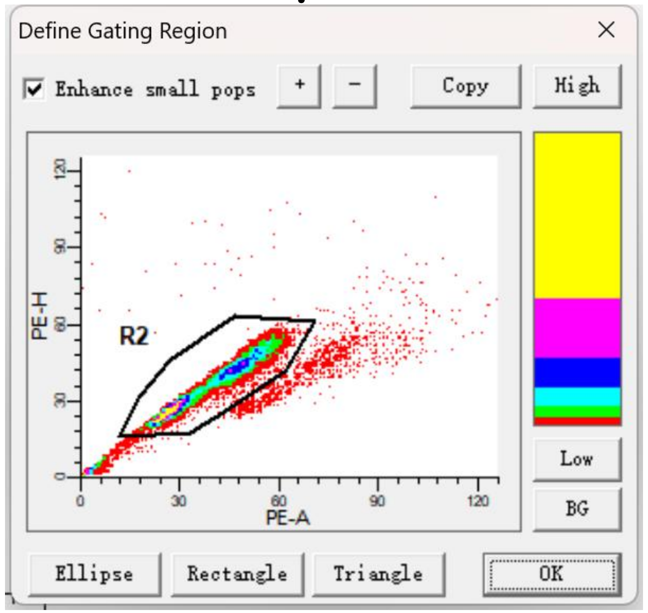

H460 4μM Gate1

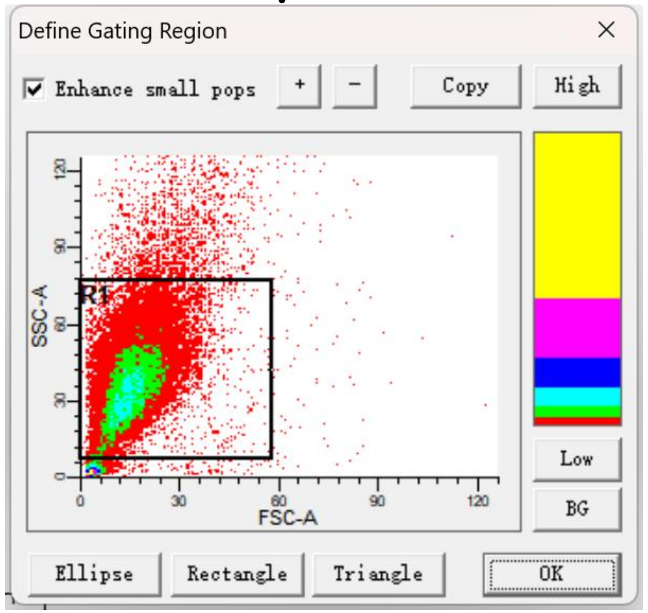

H460 4μM Gate2

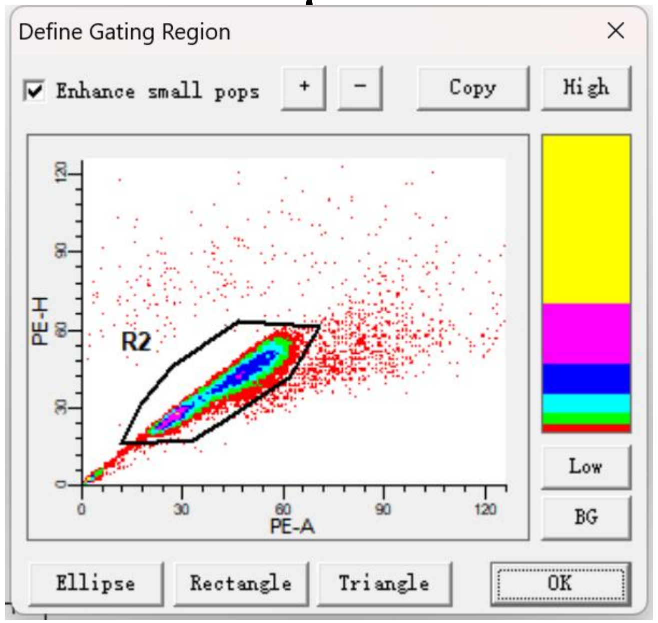

Supplement: Supplementary file 2 — Additional file 2. [file 12885_2023_10506_MOESM2_ESM.pdf]
